# Brain networks subserving functional core processes of emotions identified with componential modeling

**DOI:** 10.1093/cercor/bhad093

**Published:** 2023-03-22

**Authors:** Gelareh Mohammadi, Dimitri Van De Ville, Patrik Vuilleumier

**Affiliations:** Laboratory for Behavioral Neurology and Imaging of Cognition, Department of neuroscience, University of Geneva, CH-1202 Geneva, Switzerland; School of Computer Science and Engineering, University of New South Wales, Sydney, NSW 2052, Australia; Institute of Bioengineering, Centre for Neuroprosthetics, École Polytechnique Fédérale de Lausanne, CH - 1015 Lausanne, Switzerland; Department of Radiology and Medical Informatics, University of Geneva, CH - 1211 Geneva, Switzerland; Laboratory for Behavioral Neurology and Imaging of Cognition, Department of neuroscience, University of Geneva, CH-1202 Geneva, Switzerland; Neurology Department, University Hospital of Geneva, CH - 1211 Geneva, Switzerland; Swiss Center for Affective Sciences, Campus Biotech, University of Geneva, Switzerland

## Abstract

Despite a lack of scientific consensus on the definition of emotions, they are generally considered to involve several modifications in the mind, body, and behavior. Although psychology theories emphasized multi-componential characteristics of emotions, little is known about the nature and neural architecture of such components in the brain. We used a multivariate data-driven approach to decompose a wide range of emotions into functional core processes and identify their neural organization. Twenty participants watched 40 emotional clips and rated 119 emotional moments in terms of 32 component features defined by a previously validated componential model. Results show how different emotions emerge from coordinated activity across a set of brain networks coding for component processes associated with valuation appraisal, hedonic experience, novelty, goal-relevance, approach/avoidance tendencies, and social concerns. Our study goes beyond previous research that focused on categorical or dimensional emotions, by highlighting how novel methodology combined with theory-driven modeling may provide new foundations for emotion neuroscience and unveil the functional architecture of human affective experiences.

## Introduction

Emotions are complex and multifaceted phenomena that do not only generate rich subjective feeling states, but also powerfully impact on perception (Phelps et al. 2006), cognition (Pessoa 2008), memory (Phelps 2004; Tambini et al. 2017), and action (Damasio 2006). In spite of the lack of a general consensus on definition, various theories have been proposed to characterize emotions and their differentiation, but all remain debated and their links to specific brain processes are still equivocal (Sander et al. 2005; Meaux and Vuilleumier 2015; Adolphs 2017).

Neuroscientific approaches have mostly considered emotions either as separate entities (e.g. fear and joy) following theoretical models of discrete emotions (Ekman 1999) or instead postulated a few basic dimensions (e.g. valence and arousal) following dimensional models (Russell 2003). In both cases, the neural substrates of particular emotion categories or dimensions are usually assigned to dedicated brain areas or circuits (e.g. amygdala and striatum; Meaux and Vuilleumier 2015; Saarimäki et al. 2015). However, these approaches do not easily account for the rich variety of emotions and their anatomical overlap across distributed brain regions as shown repeatedly by neuroimaging studies in the last 2 decades (Kassam et al. 2013; Kragel and LaBar 2013, 2015; Saarimäki et al. 2015). Several meta-analyses indicate that there is no simple one-to-one association of particular emotions or dimensions with individual brain regions (Kober et al. 2008; Lindquist et al. 2012; Wager et al. 2015). Conceptual constructs of valence (Pool et al. 2016; Berridge 2019) or arousal (Satpute et al. 2019; Haj-Ali et al. 2020) do not correspond to clearly separable or unique neural substrates. Therefore, the exact role of different brain areas and their functional interaction within large-scale networks during emotional experience remains unresolved.

**Fig. 1:**
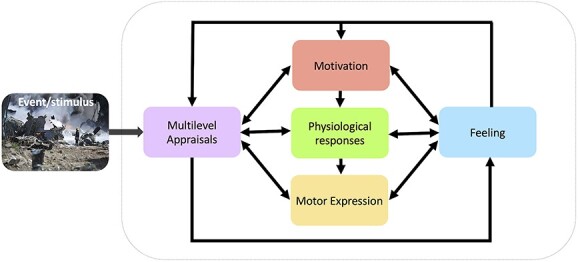
Componential model of emotion. In this framework, emotions are conceived as resulting from the concomitant (or sequential) engagement of distinct processes, responsible for the evaluation as well as the behavioral and bodily responses to particular events. According to the CPM proposed by Scherer and colleagues, from which emotion features were defined in our study, 5 distinct functional components are postulated, which can reciprocally interact to constitute an emotional experience, including appraisal mechanisms that process contextual information about the event, motivational mechanisms that promote goal-oriented behaviors and cognitions, motor expressions and physiological changes that instantiate bodily responses, and subjective feelings that may reflect an emerging component encoding conscious emotion awareness.

In contrast, other psychology theories of emotions have sought to resolve these inconsistencies by explicitly tackling the multiple ingredients of emotions. For example, the constructionist theory posits emotions as arise from conceptual categorization processes that are constructed by core affective dimensions (valence and arousal) based on interoception and integrated with other sources of knowledge determined by perception, attention, and past experiences (Barrett 2017a). This framework also helps better explain individual differences in the elicitation and neural representation of emotions, consistent with findings that emotional experiences engage multiple brain networks activated in parallel (Kober et al. 2008; Wager et al. 2015) and partly shaped by prior learning influences (Lebois et al. 2020). Another componential theory is the Component Process Model (CPM), which proposed the existence of several distinct processes that operate simultaneously and interact with each other to evaluate the meaning of events and induce adaptive changes in behavior and cognition (Scherer 2009a). This model makes an explicit distinction between specific constituents of emotions, including appraisal mechanisms, motor expression, action tendencies, peripheral autonomic changes, motivational drive, as well as various effects on cognitive and memory functions, in addition to the generation of subjective feeling states (Scherer and Moors 2019; see Fig. 1). These constituents may be shared between different emotions but engaged in different ways and to different degrees. Moreover, different appraisal mechanisms evaluate events along different dimensions, which eventually determine their personal affective significance and trigger corresponding changes in mental and bodily functions (Ellsworth and Scherer 2003). The pattern of appraisals and corresponding responses will, in turn, generate a particular emotion experience. According to such models, appraisals may encompass not only affective properties (e.g. pleasantness or valence), but also novelty, relevance to current goals, causality and agency, expectation and familiarity, control ability, as well as personal values, social norms, and other contextual factors (Sander et al. 2005).

While componential theories of emotion have been explored in detail in psychology, no study has examined how these component processes relate to particular brain networks, and which are the potential core processes commonly engaged across a range of different emotion categories. However, several neuroimaging meta-analyses results show activations in overlapping and distributed regions in response to various emotions (Kober et al. 2008; Lindquist et al. 2012; Wager et al. 2015), consistent with the notion that multiple cognitive or sensorimotor processes subserving adaptive functions may be recruited across different emotions. Such activation patterns would be consistent with componential and constructionist view suggesting that emotions activate parallel processes such as sensorimotor, physiological/interoceptive, or motivational functions, each mediated by large-scale brain networks, rather than rely on a modular emotion-specific organization. Nevertheless, previous neuroimaging studies referred to distinct functional components based on post hoc analysis or interpretation, without directly testing for such views in a theoretically driven manner. Thus, although several meta-analysis advocated for componential and constructionist approaches as a valuable framework for understanding the emotional brain (Kragel and LaBar 2016; Sander et al. 2018), to our knowledge, only 1 study (Skerry and Saxe 2015) considered specific appraisal features, a core element of componential theories, to explain brain activation patterns during emotion recognition (using verbal scenarios; Skerry and Saxe 2015). Interestingly, this study found that the appraisal feature space could better explain the neural representation of different emotions than just valence or arousal dimensions. Yet, a more comprehensive dissection of the neural underpinnings of human affective experience, going beyond the appraisal of emotion in others and including a wider range of components (e.g. motivation, expression, physiology, etc.), is still lacking.

In a search for putative neural dimensions (which we call core processes) shared across various emotions, the current study chose to focus on the CPM framework as a testbed because prior research based on this model has provided a detailed, well validated catalog of specific features and descriptors for different components (see Materials and Methods section). This makes it well-defined to be directly tested in relation to distributed brain networks. However, it is important to note that our study does not seek to prove these components or confirm the theory relative to others, but rather takes it as a presumption to reveal potential core processes using a multivariate approach.

Another key issue for emotion studies in psychology and neuroscience concerns the elicitation procedures used to induce emotional responses. Many earlier studies employed highly simplified and indirect approaches, for example, pictures of facial expressions, voices, music, or photographs that convey only a few specific emotions (Meaux and Vuilleumier 2015). These stimuli may cause a perception or recognition of particular emotions, but do not necessarily prompt a genuine emotional experience. More recent studies have therefore also explored other approaches using more naturalistic elicitation procedures (e.g. movies or memory scripts; Kragel and LaBar 2013, 2014). However, emotions are still most often studied in terms of pre-defined categories (Damasio et al. 2000) or dimensions (Nummenmaa et al. 2012), rather than functional component processes.

**Fig. 2:**
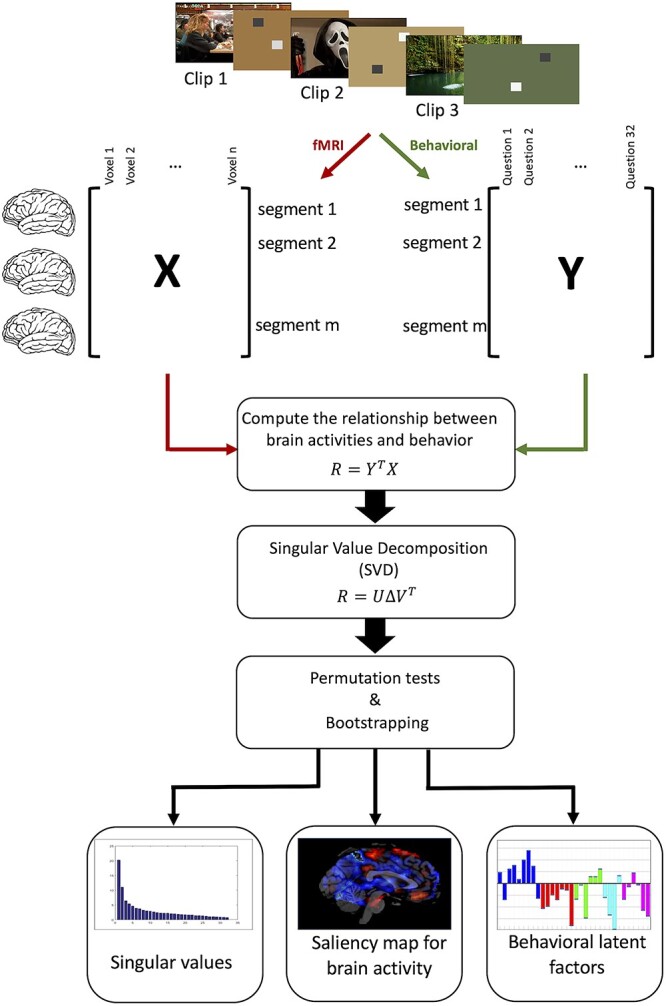
PLSC method. Participants watch emotional clips during 4 daily fMRI sessions. Matrix *X* summarizes the brain activity patterns during emotional events and matrix *Y* summarized the assessment of 32 emotion features collected during a separate behavioral session and 2 physiology features for each event. PLSC is then applied to find the commonalities between neural activity and behavioral measures. This is achieved in 3 steps, first by computing the relationship (*R*) between brain activities (*X*) and behaviors (*Y*). Then decomposing the relationship matrix *R* using singular value decomposition (SVD). And, finally, using permutation tests and bootstrapping to assess the statistical significance and saliency of latent factors.

Our study addresses these current gaps in emotion research by (i) employing a naturalistic and ecologically valid emotion elicitation procedure with a large movie data set, without assigning them to pre-defined categories, (ii) decomposing emotions into a multidimensional space organized along distinct component processes, based on participant’s experience rather than pre-defined categories, and (iii) dissecting the main “building blocks” of this space and their neural substrates using a data-driven modeling approach. Our main goal is to refine our understanding of emotion processing in the human brain through defining new methods to uncover their neural organization. We base our approach on a well-established CPM of emotion (Scherer 2001, 2009a), which provides a comprehensive representation of several key aspects of emotional behavior and experience (see Materials and Methods). Our results demonstrate how emotions may emerge from coordinated activity across a distributed set of brain networks coding for component processes associated with valuation appraisal, novelty, hedonic experience, goal-relevance, approach/avoidance tendencies, and social concerns. In doing so, our study goes beyond previous research in several ways and opens new perspectives in affective neuroscience. Figure 2 illustrates the pipeline of our approach (see Materials and Methods section for details).

## Materials and methods

This research has been approved by Geneva Research Ethics Committee (CER 09-316 and BASEC 2018-02006) and done according to the committee guidelines. Informed written consent was obtained from all participants.

### Emotion elicitation

To elicit emotions in a naturalistic and dynamic manner and track component processes, rather than presenting a sequence of unrelated stimuli assigned to pre-defined categories, we used a series of 40 movie clips that were selected and validated to cover a range of different emotions, similar to those classically investigated in psychology and neuroscience. Movies provide ecologically valid stimuli as they allow for a continuous measure of emotional responses, whose nature or intensity can be influenced by context, temporal history, or expectation (beyond just the current visual or auditory inputs). The efficacy and validity of movies has been well-established in psychological studies of emotion elicitation because of these desirable characteristics (Philippot 1993; Gross and Levenson 1995; Soleymani et al. 2008; Schaefer et al. 2010; Gabert-Quillen et al. 2015; Samson et al. 2016), but this approach remains scarce in neuroimaging research and limited to measures of basic dimensions (valence and arousal; Sabatinelli et al. 2011; Lahnakoski et al. 2012) or discrete categories of emotion (fear, sadness, etc.; Tettamanti et al. 2012; Saarimäki et al. 2015).

### Stimuli selection

To select emotionally engaging movie excerpts, we borrowed a set of 139 videos from previous researches (Gross and Levenson 1995; Soleymani et al. 2008; Schaefer et al. 2010; Gabert-Quillen et al. 2015). All excerpts were collected in both English and French languages and, matched for duration and visual quality (the original video excerpts were in English, but our experiment was in French, so we collected the dubbed version of original clips as well). We then chose video excerpts eliciting various emotions and covering different component dimensions of interest.

To this aim, we first conducted a preliminary behavioral study where emotion assessments were made in terms of discrete emotion categories, as well as according to a set of component descriptors. Discrete emotions were rated using a modified version of the Differential Emotion Scale (14 labels: fear, anxiety, anger, shame, warm-hearted, joy, sadness, satisfaction, surprise, love, guilt, disgust, contempt, calm; McHugo et al. 1982; Izard et al. 1993), whereas the componential descriptors were assessed using a questionnaire of 39 features taken from the CoreGRID instrument (Fontaine et al. 2013; see Supplementary Methods, Section A). The CoreGRID instrument includes 63 semantic concepts representing activity in the 5 major components postulated by emotion theories (appraisal, motivation, expression, physiology, feeling; see Fig. 1). Our selection concerned features appropriate to emotions experienced during movie watching where events happened to characters rather than directly to the viewer in the real-world. Based on the intensity and discreteness of categorical emotion ratings, we retained 40 videos equally covering 10 different discrete emotion categories (each predominating in 4 clips, average duration 111 s, and standard deviation of 44 s). By using several emotions (beyond the 6 basic categories), we were able to assess a more complete range of components.

The pilot ratings and validation of video clips were obtained through a web interface using CrowdFlower, a crowdsourcing platform that allows accessing an online workforce to perform a task. The selected workforce was limited to native English speakers from United States or United Kingdom and the reward was set for a minimum effective hourly wage of $6.6 (each task was rewarded ¢110 for an estimate of maximum 600 s to complete the entire paradigm); however, on average, each task was performed in ~342 s, equivalent to an average hourly wage of $11.57. The quality control of the assessments was ensured by means of ad hoc test questions about the content of the clip. Participants (*n* = 638, 358 males, mean age = 34, SD = 11) watched the full clip (on average 2.8 clips per participant), and then rated each question on how it described their feeling or experience on a 5-points Likert scale (with 1 associated to “not at all” and 5 associated to “strongly”).

### Participants in the fMRI study

Twenty right-handed volunteers (9 females, mean age 20.95, range 19–25 and standard deviation of 1.79) with no history of neurological or psychiatric disorders took part in the study. They were recruited via fliers and all native French speakers. All participants gave written consent according to the Geneva Research Ethics Committee guidelines. Demographic information (including age, sex, nationality, handedness, education, and language speaking) and Big-Five Personality Traits (using BFI-10 questionnaire; Rammstedt and John 2007) were collected prior to the experiment. There were 4 scanning sessions in total. Participants received a monetary reward of 40 CHF per session and a final bonus of 90 CHF if they completed all sessions (equivalent of 20 CHF per hour). For technical reasons, the first session of the first participant had to be discarded from the data, and another participant only completed 2 sessions out of 4. However, the remaining data from these 2 participants were used in the analysis as they included observations covering all emotion conditions.

### Experiment procedure

Participants underwent 4 testing sessions in 4 different days to complete the whole experiment. Each session started with an fMRI experiment followed by 9 behavioral assessment. At the beginning of each fMRI session, participants completed a Brief Mood Introspection Scale (BMIS) questionnaire and got prepared for the scanning. During the fMRI scanning, they watched 10 video excerpts, belonging to 10 different pre-labeled emotion categories (in random order), ensuring to probe for component processes as equally as possible across sessions. Each video was presented once only, followed by a 30-s washout clip (composed of geometric shapes moving over a fixed background, matched for average luminance and color content of the preceding clip, accompanied by neutral tones sampled from the video sound track). Participants were instructed to freely feel emotions and fully appraise the affective meaning of scenes, rather than control their feeling and thoughts because of the experiment environment (see Supplementary Methods, Section C for details).

Following each fMRI session, participants watched the same videos (whole clips) again and rated feelings and thoughts evoked by the pre-selected events in each video clip (1–4 excerpts per clip, based on salient events identified in a separate study, total 119 excerpts), according to how they felt when they first saw it inside MRI scanner. Participants were explicitly advised to reflect on their own feelings and thoughts and not what is intended to be felt in general by watching the same event. Ratings were obtained for pre-selected events with perceptually and/or emotionally salient content (between 1 and 4 events per each video, with mean of 2.9 and duration of 12-s per event). This allowed us to ensure ratings corresponded to a precise event, and not more global judgments about the video. Answers were given immediately after watching the emotional event by pausing the video. Emotional events were selected from a separate pilot experiment by other subjects (*n* = 5) who made continuous evaluations with CARMA (software for Continuous Affect Rating and Media Annotation; Girard 2014) allowing second by second ratings (see Supplementary Methods, Section B).

For post-fMRI ratings, participants had to choose 1 or 2 of the emotion labels (primary and secondary most felt) from the list of 10 discrete emotion categories (fear, anxiety, anger, joy, sadness, satisfied, surprise, love, disgust, and calm), and to rate each of 32 CoreGRID features selected in our pilot study using a 7-point Likert scale (1 corresponding to “not at all” and 7 corresponding to “strongly”). Seven other features from the CoreGRID list concerned some of the physiological and expression measures were not included in the current analyses. Each fMRI session and 10 behavioral session lasted for about 1 and 2 h, respectively (about 3 h $\times$ 4 sessions = 12 h of experiment for each participant), including preparation time. Behavioral rating sessions were performed on a separate PC in a quiet room. In total, 2,276 video events were rated along the 32 GRID dimensions described above (see Supplementary Methods, Section D for details).

### Data acquisition

MRI was performed on a 3T Siemens TIM Trio scanner at the Brain and Behavior Laboratory of the University of Geneva, with a 32-channel head coil using gradient-echo T2^*^-weighted echo-planar image sequence for functional images (TR = 2,000 ms, TE = 30 ms, flip angle = 85°, FOV = 192 mm, resolution = 4 × 64, 35 axial slices, voxel size 3 × 3 × 3 mm). High-resolution T1-weighted structural images and susceptibility-weighted images were also collected. Each video was presented during a separate MRI acquisition run to ensure independence between different stimuli. The acquisition time for each run was about 164 s on average. Stimuli presentation and rating were controlled using Psychtoolbox-3, an interface between MATLAB and computer hardware. During the fMRI session, participants watched the stimuli on an LCD screen through a mirror mounted on the head coil. The audio stream was transmitted through MRI-compatible Sensimetrics Insert earphones (model S14). Peripheral physiological measures were also collected during the fMRI session including heart activity, respiration, and electro dermal activity (EDA) using BIOPAC system. However, because of technical reasons, the EDA was missing for several sessions in some subjects and so these data were excluded from subsequent analysis of fMRI data to keep as many sessions/subjects as possible in the brain analysis (In a separate study with a distinct analysis focusing only on physiology and behavioral data (Menétrey et al. 2022) where the total number of variables was much smaller than the current fMRI study, we removed all sessions with missing EDA and instead retained EDA as a variable of interest in our analysis.). Similarly, to detect facial expressions such as smiles and frowns, we recorded electromyogram (EMG) during the full scanning sessions, but because of electromagnetic interference from other devices and poor signal overall, facial motor activity could not be reliably retrieved and was not analyzed either.

### Preprocessing of fMRI data

Initial processing of the fMRI data was performed using Statistical Parametric Mapping 12 (SPM12) software (Wellcome Department of Cognitive Neurology, London, UK). The data were corrected for slice timing and motion, co-registered to high resolution structural images, and normalized to Montreal Neurologic Institute (MNI) space. Spatial smoothing was applied at 6 mm and temporal data were high-pass filtered at 0.004 cutoff point. No physiological noise correction was applied because physiology covariates are assumed to constitute one of the constituents of the CPM and therefore have to be retained in the analysis. Changes in neural activation were modeled across the whole brain using the general linear model (GLM) as implemented in SPM12. For each run, the blood-oxygenation-level-dependent (BOLD) signal was modeled using multiple regressors, 1 per each emotional event (without overlap) plus 1 representing the washout period, which were convolved with the hemodynamic response function (HRF). The onset of each HRF is aligned with the beginning of each emotional events. Six motion parameters (translations in $x$, $y$, and $z$, roll, pitch, and yaw) were also added to account for nuisance effects. No mask was applied to the GLM estimations and data from whole brain were used in the later analysis. For each emotional event, a contrast map between the emotional event and washout *β*s was computed and then used as the differential neural marker of the corresponding emotional experience(s) associated with this event. We also analyzed the framewise displacement using 2 metrics (Power et al. 2014; Huijbers et al. 2017) with the standard thresholds of 0.5 and 0.2 mm, respectively, as suggested by other literatures. Our analysis revealed that only 5 runs out of 770 to have more than 20% displaced frames. However, removing the affected 5 runs did not change the final results, significantly.

### Physiology signal preprocessing

All physiology signals (heart pulsation, respiratory, and EDA) were acquired throughout the scanning sessions at 5,000 Hz sampling rate. To preprocess this data, we used AcqKnowledge 4.2 and MATLAB. Signal artifacts and signal losses were corrected manually using Endpoint function in AcqKnowledge software, which interpolates the values between 2 selected points. Then signals were downsampled to 120 Hz and a comb-pass filter was applied to remove the scanner artifacts. The heart activity signal was filtered with a band-pass filter between 1 and 40 Hz and the heart rate (HR) was computed using peak detection technique and was converted to beats per minute. Results were controlled to make sure that the estimated HR is in the normal range of 60–100 beats per minute and otherwise corrected manually. All automatically detected peaks were verified visually and any misdetection was corrected manually. The respiration signal was band-pass filtered between 0.05 and 1 Hz and similar to heart signal, respiration rate (RR) was estimated. The EDA signal was also processed, but finally discarded from analyses because of a large portion of missing values. Average HR and RR (after orthogonalizing them to head motion vectors) were computed for each emotional event and then treated similarly as other GRID descriptors to assess neural effects associated with increases or decreases of HR and RR. Please note we did not include physiology signals as nuisance regressors in the fMRI analysis (e.g. RETROICOR) in order to minimize the risk of removing potential signals of interest (based on theoretical predictions from CPM), while still cleaning the fMRI data from potential noise associated with brain motion (using head realignment parameters). We also verified that the head motion-related regressors were unlikely to remove concomitant effects on neural activity associated with peripheral physiology by ruling out any systematic correlation between head motion and HR or RR [mean correlation *r* = −0.015 across all movie runs; not significant when tested across participants (*n* = 20, *P* = 0.802), movie clips (*n* = 40, *P* = 0.723), or single runs (*n* = 770, *P* = 0.134)].

### CPM and CoreGRID

We based our analysis of behavioral and fMRI data on a previously established model (Scherer 2001) assuming 4 different functional components, including (i) a motivational component that causes changes in action tendencies, (ii) an expression component that implicates changes in motor behavior and action, (ii) a physiological component that corresponds to changes in peripheral autonomic activity, and finally, (iv) a feeling component that reflects the conscious experience concomitant to changes in all other components (Scherer 2009b). A series of 144 descriptors for distinct features of each of these components has been defined in the GRID instrument (Fontaine et al. 2013) in order to cover various emotional experiences according to their frequent use in the literature, cross-cultural adaptability, and common occurrence in self-reports. In our study, we used the CoreGRID instrument, a validated brief version of the GRID encompassing 63 semantic items (Fontaine et al. 2013), among which we selected those applying to our experimental paradigm and compatible with a third-person perspective of emotional events. These features and their links with main components are listed in Table 1.

**Table 1:** Items of GRID questionnaire. The left column shows the list of the 32 GRID items rated by participants for each of the 119 selected events in movies and 2 peripheral physiology measures (marked with alphabet). In this column, the abbreviation “s.th.” stands for “something.” The second column represents the component that each GRID item belongs to. The 6 right columns correspond to the 6 factors obtained from a factorial analysis (D1–D6) and their loading coefficients. Loading coefficients with maximum values for each factor are color-coded to help interpretation and group them in terms of corresponding factors: D1 can be interpreted as valence, D2 corresponds to arousal, D3 mainly encodes motor expression and bodily changes, D4 represents novelty, D5 relates to action tendencies, and finally D6 represents norms. Coefficients highlighted in bold are statistically significant (*P*-value < 0.05).

**Questions:** (While watching this scene, did you…)	**Components**	**D1**	**D2**	**D3**	**D4**	**D5**	**D6**
Feel good?	Feelings	**−0.85**	−0.12	−0.17	−0.06	−0.13	−0.03
Feel situation was unpleasant for you?	Appraisal	**0.81**	0.20	0.29	0.01	0.10	0.11
Feel bad?	Feelings	**0.81**	0.27	0.25	0.01	0.09	0.06
Want to undo what was happening?	Motivation	**0.81**	0.21	0.15	0.01	0.15	0.17
Want the situation to continue?	Motivation	**−0.81**	0.12	−0.05	−0.07	−0.11	−0.02
Feel the urge to stop what was happening?	Motivation	**0.81**	0.19	0.17	0.00	0.16	0.16
Feel it was unpleasant for someone else?	Appraisal	**0.71**	0.13	0.07	0.18	0.16	0.18
Feel calm?	Feelings	**−0.69**	−0.19	−0.26	−0.11	−0.18	−0.08
Feel strong?	Feelings	**−0.54**	0.04	−0.08	−0.08	−0.01	0.03
Want to tackle the situation and do s.th.?	Motivation	**0.51**	0.35	0.00	−0.05	0.38	0.14
Feel an intense emotional state?	Feelings	0.19	**0.82**	0.28	−0.07	0.06	0.06
Experience an emotional state for a long time?	Feelings	0.17	**0.79**	0.24	−0.06	0.06	0.04
Feel motivated to pay attention to the scene?	Motivation	−0.15	**0.42**	−0.08	0.06	0.08	0.05
Have a feeling of lump in the throat?	Physiology	0.42	**0.40**	0.29	−0.09	0.14	−0.02
Show tears?	Expression	0.15	**0.39**	0.01	−0.12	0.08	−0.13
Think it was important for somebody’s goal need?	Appraisal	0.06	0.18	0.06	0.01	0.00	0.03
Experience muscles tensing?	Physiology	0.45	0.21	**0.49**	0.01	0.22	0.04
Produce abrupt body movement?	Physiology	0.19	0.10	**0.48**	0.10	0.19	0.09
Close your eyes?	Expression	0.23	0.05	**0.45**	0.00	0.05	0.01
Have stomach trouble?	Expression	0.35	0.37	**0.45**	−0.01	0.10	−0.04
Feel warm?	Physiology	0.02	0.15	**0.39**	−0.02	0.10	−0.05
Have eyebrow go up?	Expression	0.15	0.04	0.30	0.27	0.07	0.20
Press lips together?	Expression	0.26	0.24	0.28	0.01	0.05	0.05
Have the jaw drop?	Expression	0.06	0.09	0.23	0.26	0.10	0.12
^a^Heart rate	Physiology	−0.01	0.02	0.20	0.03	−0.06	0.02
^a^Respiratory rate	Physiology	0.09	−0.04	0.15	0.05	0.05	0.06
Feel that the event was unpredictable?	Appraisal	0.08	−0.02	0.09	**0.77**	0.01	0.06
Feel the event occurred suddenly?	Appraisal	0.21	0.03	0.13	**0.68**	0.05	0.06
Think that the consequence was predictable?	Appraisal	0.06	0.07	0.02	**−0.42**	0.09	0.05
Think the event was caused by chance?	Appraisal	0.01	−0.04	−0.01	**0.42**	−0.04	−0.01
Want to destroy s.th.?	Motivation	0.30	0.15	0.20	−0.06	**0.73**	0.05
Want to damage, hit or say s.th. that hurts?	Motivation	0.35	0.18	0.18	−0.06	**0.70**	0.12
Think it violated laws/social norms?	Appraisal	0.59	0.07	0.16	0.09	0.18	**0.64**
Think it was incongruent with your standards?	Appraisal	0.61	0.06	0.20	0.06	0.16	**0.62**

### Hierarchical clustering

To analyze similarity/dissimilarity between discrete emotions in terms of their profile of component features, we applied a hierarchical clustering analysis. To define this componential profile, we computed the average value of CoreGRID items for each discrete emotion class (emotion class of each rating was based on its primary emotion category), which represents the class centroids. Hierarchical clustering allows for grouping similar items into 1 cluster and merges pairs of clusters as it moves up the hierarchy. One advantage of such algorithm is the possibility to interpret the similarity at different levels. Here, we used squared Euclidian distance as the similarity measure between clusters (Ward’s method).

### Exploratory factor analysis

We applied factor analysis to all GRID items from all 4 components to find the underlying commonality across different items, allowing us to compare our data set with previous work using similar analyses (Fontaine et al. 2007). Based on Kaiser’s criterion, 6 factors were selected (see section Underlying Factors), which accounted for 48% of total variance, and applied orthogonal varimax rotation to simplify the expression of a particular factor in terms of just few major items. The interpretation of each factor is based on its relationship with specific GRID item set.

### Partial least square correlation

To identify consistent patterns of covariations among component features and concomitant changes in brain activity patterns, we employed Partial Least Square Correlation (PLSC), a multivariate statistical modeling technique that extracts the commonalities between neural activity and behavior through an intermediate representation of latent variables (LV; Krishnan et al. 2011). In this method, response and independent variables are projected to a new space of LV, such that the latter has the maximal covariance. Here, we included the behavioral ratings on all 32 CorGRID items plus 2 physiology measures (HR and RR) on one hand, and fMRI data from whole brain obtained for all emotional events across all movies (*n* = 2,276) on the other hand. In this analysis, BOLD activity from $V$ voxels is stored in matrix ${X}_{M\times V}$ with $M$ rows as the number of observations, and ratings of emotion experience are stored in matrix ${Y}_{M\times N}$ where $N$ is the number of behavior descriptors. Columns in both $X$ and $Y$ are normalized to within subject *z*-scores. The relation between $X$ and $Y$ is stored in a correlation matrix $R$ as $R={Y}^TX$, which is then submitted to a singular value decomposition (SVD) to obtain 3 different matrices as $R=U\Delta{V}^T$, where $U$ and $V$ represent saliences values (loadings) for $Y$ and $X$, respectively. LV are computed as ${L}_X= XV$ and ${L}_Y= YU$that model the relationship between BOLD signal and behavioral data (Abdi and Williams 2012). We assessed the significance of LV with permutation tests (Efron and Tibshirani 1986; 1,000 iterations) and LV with *P*-value < 0.01 were retained for interpretation.

We also verified our PLSC results in terms of statistical significance and reliability using independent methodology based on permutation tests and bootstrapping, respectively. Because standard cross-validation techniques or power analysis are not best applicable to the PLSC method, the optimal sample size and generalizability of results cannot be calculated solely based on the number of subjects or model parameters, but should instead be tested based on the precision of the model estimation and its standard error (Marcoulides and Chin 2013). A robust method to assess the precision of estimates is through resampling methods like Monte Carlo simulation, with the most popular approach based on bootstrap sampling (Efron and Tibshirani 1986). In our study, we took a conservative bootstrap approach, in which we limited the number of subjects used at every iteration of bootstrap to be ~70% of participants (two-thirds of sample). This guarantees that data from ~30% of the subjects are excluded at every iteration and the estimates are solely based on the resampled data from a portion of subjects. As can be inferred, this method also implicitly examines the generalizability of the method to different set of samples and allowed us to estimate the robustness of our results. To do so, for each bootstrap sampling iteration, the PLSC procedure was repeated [on a set of 14 randomly selected subjects (about 70% of the subjects)] and the variance of each element of each LV was computed over 1,000 iterations. The stability scores ${S}_{u_i}$ and ${S}_{v_i}$ for the $i$th elements of factors $u$ and $v$ are obtained as ${S}_{u_i}=\frac{u_i}{\sigma \left({u}_i\right)}$and ${S}_{v_i}=\frac{v_i}{\sigma \left({v}_i\right)}$, where $\sigma \left({u}_i\right)$ and $\sigma \left({v}_i\right)$ denote standard errors for ${u}_i$ and ${v}_i$ where $\sigma \left({u}_i\right)$ and $\sigma \left({v}_i\right)$ denote standard errors for ${u}_i$ and ${v}_i$, respectively. Stability scores higher than 2.5 or lower than −2.5 (corresponding to *P* < 0.01) were considered as significant, indicating voxels that reliably respond to a particular condition. Because resampling methods can cause axis rotation and alter the order of LVs, we used Procrustes rotation (Ten Berge 1977) to correct for such effect. This approach allowed us to identify voxels with highest loadings for each LV, and emotion features contributing most to each of these LVs. Results thus delineate distinct brain-wide networks and the component feature profiles associated with their activation, presumably underlying different dimensions of emotion over various episodes. Importantly, this patterning is obtained in a purely data-driven manner, without assigning features to particular components or appraisals, and without grouping them according to pre-defined discrete emotion categories. Please also note that our participant sample size is similar to other studies exploring social appraisal features with a smaller stimulus data set (Skerry and Saxe 2015) and much larger than in other recent work using machine learning approaches with a similar comprehensive video data set (e.g. *n* = 5 participant in Horikawa et al. 2020).

In addition, we ran a K-fold cross-validation technique, though this is not standard for PLSC, in order to further test for the generalizability and reliability of model using a different perspective (similar to machine learning methods). This procedure yielded very similar results that are reported in the Supplementary Results, Section B, and further supported the generalizability of our PLSC findings.

## Results

As described above, the main part of our study presented a group of healthy volunteers (*n* = 20) with a series of 40 video clips, which were selected from a large data set validated in a preliminary study so as to convey a large range of emotions. Participants watched these video clips while whole-brain activity was measured with fMRI and peripheral physiology was monitored through HR, RR, and EDA. Movies were presented over 4 different sessions on separate days (10 movies in each, total duration = 74 min). During the movies, participants were encouraged to get absorbed in the scenes and let their emotions freely flow without any particular task. After each session, they were presented with the same videos where particular excerpts containing a salient or emotional event (between 1 and 4 excerpts for each clip) were highlighted for assessment following a video pause. Participants had to rate these events in terms of several dimensions of emotional experience (using a 7-point Likert scale). These ratings included (i) a series of 32 descriptors corresponding to major emotion features identified by the CPM (GRID items, see Table 1) and validated by previous psychology research across several cultures (Fontaine et al. 2013) and (ii) a list of 10 discrete emotion categories that could occur during movies (fear, anxiety, anger, sadness, disgust, joy, satisfaction, surprise, love, and calm). We subsequently used the 32 emotion features and 2 peripheral physiology measures (HR and RR) to identify consistent covariations corresponding to coordinated patterns of emotional responses, and then applied a multivariate modeling approach to relate each of these patterns to distinctive brain network activations. Because of high noise and frequent missing values in some subjects, EDA was excluded from the final data set in order to retain all participants for the fMRI analysis. Finally, we also examined how the different components identified in our model were modulated according to discrete emotion categories and dimensions postulated in classic theories.

**Fig. 3:**
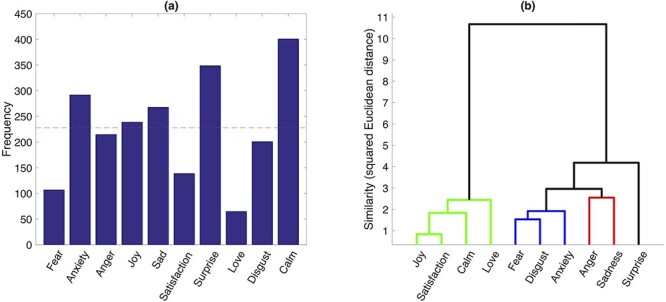
Histogram and hierarchical clustering of discrete emotions. a) Histogram of categorical emotions based on their frequency in the ratings of 119 emotional event by 20 participants (data from 2 participants were not complete). The red dashed line shows the ideal frequency if samples were distributed uniformly. b) Hierarchical clustering of the discrete emotion profiles in the GRID space using Ward algorithm. The higher-level clusters distinguish between positive and negative emotions. The lower-level clusters reflect a segregation of feelings in terms of pleasantness (green), surprise (black), distress (blue), and annoyance or frustration (red).

**Fig. 4:**
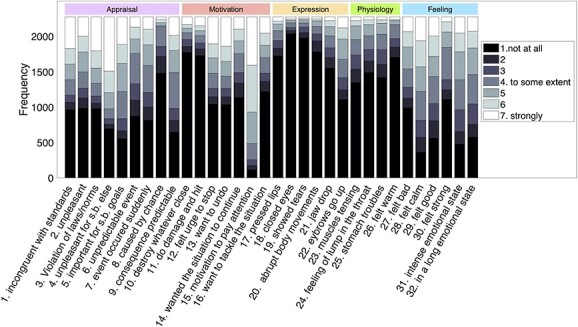
Histogram of GRID items. Histogram of ratings for all the 32 GRID items based on the number of times a specific rating (1–7) was selected across all assessments (119 assessments per participant) and all 20 participants (the data from 2 participants were not complete). The abbreviation “s.b.” stands for somebody.

### Behavioral and physiological measures

To illustrate the variability of elicited emotions, Fig. 3a presents the histogram of the discrete emotion categories, selected by our participants as the most dominant emotion during each of the salient movie excerpts. Although the distribution of discrete emotions is not uniform, it shows that, except for love, our stimulus material and experimental design was successful in eliciting a wide range of different emotions, allowing us to obtain a comprehensive survey of the componential space. The nonuniform distribution of emotion categories (unlike results from the movie selection phase; see Materials and Methods) is because of the fact that these ratings concerned short emotional episodes (single events) and were obtained from a varying number of segments across different video clips (unlike the more global judgments made for whole movies during preselection). Moreover, single events in a movie did not necessarily elicit the same emotion as the global judgment made for an entire movie clip, which highlights the importance of using and characterizing short segments for fMRI analysis.

In addition, a histogram count of the GRID features across movie emotional events (Fig. 4) showed that ratings of all 32 items were generally well spread across the 2 ends of the continuum, with only a few items exhibiting a distribution skewed toward the lower end (rated as 1). This bias was more evident for features from the expression component, most likely because of the passive condition of emotional experience during movie watching that does not require any direct behavioral responses or communication.

Altogether, these behavioral data demonstrate that our procedure could successfully cover the whole componential space for a range of different emotions and thus effectively test for patterns of shared variability across the different stimulus conditions.

**Fig. 5:**
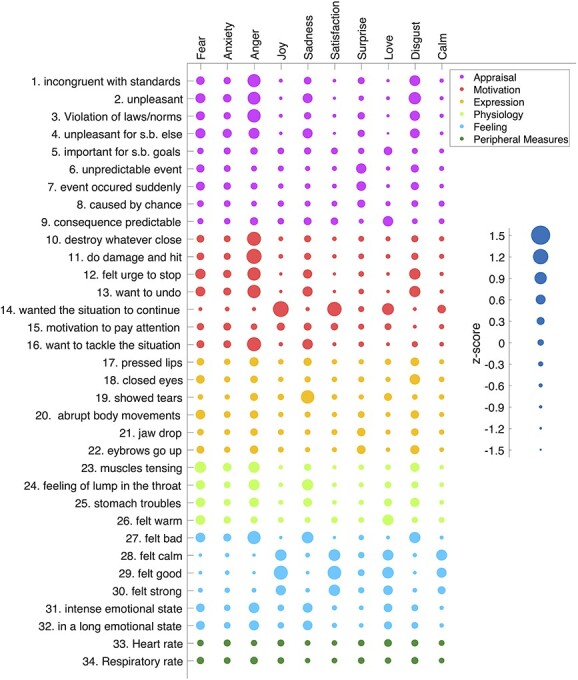
Discrete emotion profiles in GRID space. Average profile of each discrete emotion on the 32 GRID features and 2 peripheral physiology measures (HR, RR) after within-subject normalization. For each discrete emotion, all assessments from all 20 participants with that discrete emotion label were used. Each bubble corresponds to a *z*-score using an exponential scaling. The smallest bubble represents a *z*-score = −1.27, corresponding to “not at all,” and the biggest bubble represents a *z*-score = +1.23 corresponding to “felt strongly.” Colors represent the different emotion components to which GRID items belong to.

Finally, physiology recordings obtained during movies (see Materials and Methods) confirmed significant bodily changes across different emotions for HR $[F(9,2291)=3.767,P<0.001,{\eta}^2=0.014)]$, RR [$F(9,2291)=3.767,P<0.001,{\eta}^2=0.014$], and tonic EDA $[F(9,2029)=3.415,P<0.001,{\eta}^2=0.014)]$. No reliable effect was found for phasic EDA $[F(9,2029)=1.237,P=0.268,{\eta}^2=0.005)]$. Please note that these physiology measures were acquired during fMRI as objective indices to further assess the GRID physiology component, as assumed in CPM, without relying only on subjective reports of physiological changes that might be less sensitive. We did not compute more sophisticated characteristics of physiology parameters (e.g. variability) as in some behavioral studies (Kragel and LaBar 2013), since such indices were not part of the GRID descriptors and reliable physiological data are difficult to record during concomitant fMRI. However, in a separate analysis, we found that the absolute mean and variance of HR, RR, and EDA could predict appraisal GRID descriptors significantly above chance level, whereas they did not reliably predict discrete emotion ratings (Menétrey et al. 2022). These findings suggest that our measures were robust enough to be used as GRID descriptors in subsequent fMRI analysis, but insufficient to fully differentiate complex emotional experiences by themselves (i.e. their information may already be captured by other GRID descriptors; see Materials and Methods and Supplementary Methods, Section A). To fully characterize emotional responses in terms of physiology processes, more analyses should therefore be conducted, which is out of the scope of this study (see Kragel and LaBar 2013). A more comprehensive analysis of the relationship between GRID items, physiology recordings, and discrete emotion ratings is presented in Menétrey et al. (2022).

### Cluster analysis

To analyze the relation among different emotion categories within the componential model space, we performed a hierarchical clustering analysis on the average profile of each discrete emotion along 32 GRID features plus 2 peripheral physiology measures of HR and RR. As can be seen in Fig. 5, different features were present to different degrees for different discrete emotions, whereas several features were shared by more than 1 particular emotion. Accordingly, the clustering results indicated a clear distinction between positive versus negative emotions at the higher level of differentiation (Fig. 3b), whereas 4 different clusters were observed at the lower level representing, respectively, pleasant feelings (joy, satisfaction, love, and calm), distress (fear, disgust, and anxiety), annoyance (anger and sadness), and surprise. Surprise showed a higher similarity to negative categories than to pleasantness, possibly reflecting that it came mostly with negative contexts in our movie data set.

Altogether, these findings demonstrate that theoretically meaningful clusters can be derived from our set of GRID features, fully in accordance with emotional dimensions commonly considered in neuroscience studies. This, in turn, further validates our experimental paradigm by showing it could successfully elicit different emotion categories with expected characteristics.

### Underlying factors

For completeness and comparison with prior psychology research (Fontaine et al. 2007), we also performed an exploratory factor analysis to recover common dimensions underlying the different GRID features (Fontaine et al. 2013). Table 1 summarizes these 6 factors and their relative loadings. Overall, about 47.8% of the total variance was explained by these 6 factors. The GRID items most strongly associated with these 6 factors suggest they may be linked to pleasantness (21.6%), arousal (7.6%), expression (5.9%), novelty (5.0%), action tendency (4.6%), and social norms (3.1%), respectively. This accords with previous findings obtained with similar component models (Fontaine et al. 2007). These results also confirm that emotional experiences involve more than 2 dimensions of valence and arousal when explored across a variety of conditions with ecological validity.

Taken together, these behavioral data converge to indicate that a comprehensive differentiation of emotions would require not only to go beyond valence and arousal dimensions, but also to better characterize the commonality and specificity of different types of emotions. Accordingly, this also implies that mapping the neural underpinnings of emotion requires a multivariate approach that transcends valence-arousal representations or simple oppositions between discrete categories, as we propose with the componential approach in the next section.

### Decomposing emotions into functional core processes and their neural substrates

To determine how component processes of emotion are organized in relation to functional brain systems, we collected fMRI data from our 20 healthy participants while they watched the 40 emotional video clips, and then analyzed BOLD time-courses to identify patterns of brain activity corresponding to systematic covariation in their ratings of specific componential features during the movies (see Fig. 2). For each participant, we used ratings of each of the 32 GRID features for each of the 119 salient emotional events selected from these movies (total of 2,276 surveys, including 72ʹ832 rating scores), plus the HR and RR *z*-score values for these events.

To decompose emotion responses into core functional processes, we applied a multivariate technique, namely PLSC (Krishnan et al. 2011), enabling us to analyze covariance in 2 feature spaces: (i) the multidimensional structure of emotion ratings along all GRID items (CPM), and (ii) the multidimensional activation patterns across all brain voxels. PLSC identifies a set of orthogonal LVs for each space that express maximum cross-covariance and thus represent shared information in the 2 spaces (i.e. behavior and brain). In other words, this method allows for modeling the functional relationship between the coordinated mobilization of multiple emotion features and corresponding variations in neural activity.

After preprocessing fMRI data according to standard pipelines and normalizing behavioral ratings to within-subject *z*-scores, the PLSC analysis was applied to the whole sample from our 20 participants. Statistical significance of components (*P*-values) was calculated using permutation tests, and *z*-scores reflecting reliability of loadings (a.k.a., saliencies) were estimated using bootstrap ratio. To determine the generalizability of the method and overcome limited sample issues, a conservative bootstrap strategy was taken to further probe for data robustness across individuals whereby, at every bootstrap iteration, only 14 randomly selected subjects were used (i.e. one-third of the sample was excluded), and the final behavioral loadings at the full group-level were then estimated as the average loadings across all bootstrap iterations. The standard deviation of each loading was considered as a measure of stability of the loadings across the different subsets of subjects and hence provided an estimate for the robustness/generalizability of our results (see Materials and Methods for details). In addition, we also performed a K-fold cross-validation which produced very similar results (see Supplementary Results, Section B).

**Fig. 6:**
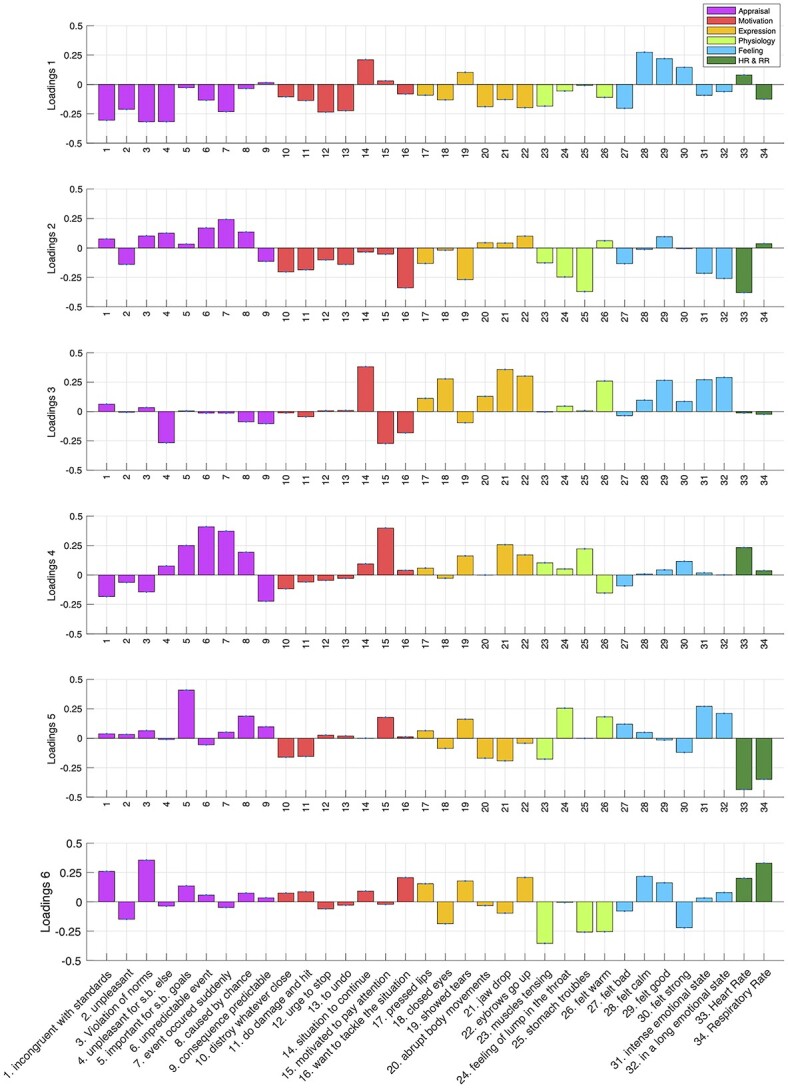
Loadings of PLSC. Loadings of PLSC for GRID items (behavioral) and peripheral measures corresponding to the 6 significant LV (1–6), respectively, interpreted as: valence, novelty, hedonic impact, goal monitoring, goal relevance, and avoidance. Each loading vector corresponds to 1 brain activity map that is shown in Fig. 7. The blue error bars indicate the standard deviation for each value that reflects the reliability of the loading when apply bootstrapping; however, because of very small variation, they are not very visible.

The PLSC analysis across all GRID items and physiology measures revealed 6 significant LV with *P* < 0.01. These LVs represent distinct combinations of behavioral features with concomitant brain patterns. Importantly, please note that LVs are defined in terms of both their component features and associated neural activity. Figure 6 shows the loading profile along all GRID and physiology features for each LV identified here: LV1 (*P* < 0.001, 19.0%), LV2 (*P* < 0.001, 11.0%), LV3 (*P* < 0.001, 6.0%), LV4 (*P* < 0.001, 5.1%), LV5 (*P* < 0.01, 4.6%), and LV6 (*P* < 0.01, 3.4%); numbers in parenthesis indicate *P*-value and percentage of covariance explained, respectively. Positive or negative loadings of particular features reflect the relative presence or absence of these features, respectively. Their weighted sum represents a specific combination linked to a particular brain activation pattern, whose expression characterizes a given functional core process (FCP) underlying the generation of an emotion response. All maps were thresholded at positive or negative saliency values of >2.5 or < −2.5 (*P* < 0.01).

**Fig. 7:**
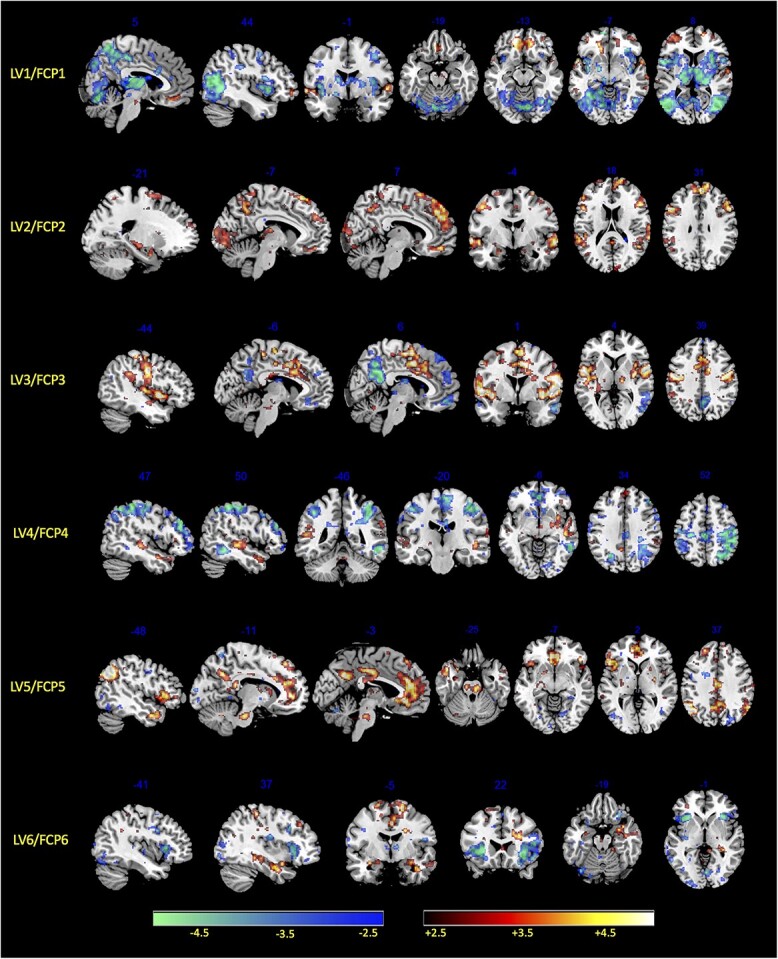
Brain saliency maps. Brain activity maps of relative saliencies corresponding to each of the 6 significant FCP, a.k.a. LV, obtained by the PLS analysis of GRID ratings. The red spectrum accounts for positive saliencies above +2.5 and blue spectrum corresponds to negative saliencies below −2.5.

The dominant behavioral profile of LV1 shows higher weightings for several features related to the appraisal of values with motivational aspects and the valence dimension of the feeling component. As illustrated in Fig. 6, positive ratings for this LV reflect events with low/no unpleasantness, low/no incongruence with standards, a desire to continue/not to stop, and feeling good/not feeling bad (among others). On the brain side, the voxel-wise saliency map of LV1 exhibits significant positive weights (>2.5) in ventromedial prefrontal cortex (VMPFC), lateral orbitofrontal cortex (OFC), central/lateral amygdala, and ventral tegmental area (VTA), and significant negative weights (<−2.5) in anterior and dorsal insula, mid-cingulate cortex, thalamus, basal ganglia (caudate and putamen), dorsal amygdala and substantia innominata, as well as medial parietal and lateral occipital areas (Fig. 7).

The second latent variable (LV2) shows higher weightings on items related to appraisals of unexpected event and detection of changes (i.e. sudden and unpredictable, with brief emotion intensity), a pattern that can be interpreted as novelty detection (Fig. 6). The corresponding brain saliency maps show significant positive weights bilaterally in dorsomedial prefrontal cortex (DMPFC) and dorsolateral prefrontal cortex (DLPFC), inferior frontal gyrus (IFG), posterior cingulate cortex (PCC), together with large effects in sensory (auditory and visual) cortices, as well as smaller activation clusters in dorsal amygdala, hippocampus, and parahippocampal gyrus (PHG). Negative saliency weights are much weaker and limited, involving only very small parts of anterior insula and rostral anterior cingulate cortex (ACC; Fig. 7).

The third latent variable (LV3) loads mainly on expression-related features (such as closing the eyes, pressing the lips, raising eyebrows) along with feeling features related to pleasantness and arousal (warm, good, intense, and lasting experience), which together might encode generally pleasurable sensation and hedonic impact. This LV exhibits significant positive saliencies in widespread sensorimotor areas, including the primary somatosensory and motor cortices, particularly over the central fronto-parietal operculum (face area), but also SMA, preSMA, dorsal ACC, posterior insula, left ventral pallidum, VTA, and brainstem (central pons). Negative saliencies were again weak, essentially limited to the PCC, inferior parietal lobule (IPL), and a small sector of VMPFC, which may constitute parts of the default mode network (DMN; Raichle 2015).

The latent variable LV4 unfolds mainly on appraisal components related to expectation and goal settings, as well as motivated attention and congruence with norms, without any consistent loadings related to feelings. These features may reflect encoding of goals and intentions (of someone else in movies) or violations of expectations. At the brain level, LV4 is associated with positive saliency weights predominating in STS and temporal pole, as well as in DMPFC and precuneus, partly overlapping with social cognition and theory-of-mind networks (Amodio and Frith 2016). There were also smaller clusters in the temporoparietal junction, IFG, and pallidum, all parts of the ventral attention orienting network (Corbetta and Shulman 2002). Widespread negative saliency weights were found in superior parietal cortex (intraparietal sulcus), DLPFC, ACC, and posterior SMA, overlapping with dorsal attention networks.

The next significant latent variable LV5 loads selectively on appraisal of event relevance for someone else with some features of physiology (warmth and lump in the throat) and feeling components (high intensity but no valence). There is also a relative lack/suppression of aggressive motivational tendencies (wanting to attack or damage), which, altogether, may reflect a state of prosocial concern or caring for others. The brain saliency map reveals a strong positive correlation with areas of the DMN, particularly MPFC and PCC, but also smaller clusters in bilateral anterior insula, inferior parietal lobe, caudate, and brainstem (possibly overlapping with locus coeruleus). There are no consistent negative weights.

Finally, the last significant latent variable LV6 loads on antisocial features (e.g. incongruence with norms), attention and approach/attack, but also some degree of pleasant feelings (good or calm), which might relate to curiosity/interest and elements of aggression or active defense. The corresponding brain saliency map shows positive weights in the amygdala, VTA, medial OFC/VMPFC and superior frontal gyrus (SFG), as well as somatosensory areas and PCC (Fig. 7), whereas negative saliencies were mainly found in the bilateral anterior insula, lateral OFC, rostral ACC, and posterior visual areas (Fig. 7). A summary of average saliency values across cortical areas for each LV is provided in Supplementary Results, Section A, Supplementary Table 3.

The 6 LVs also disclosed distinct patterns of loadings for the peripheral physiology measures (Fig. 6, last 2 columns, dark green). Both HR and RR exhibited negative weights for LVs 5 and 6, whereas LV2 was associated with negative weights and LV4 with positive weights of HR alone (not RR). Both measures were only weakly modulated by LVs 1 and 3. These physiology patterns seem compatible with psychological processes putatively associated with each LV (Harrison et al. 2013). However, similar LVs were obtained when repeating our PLSC analysis without physiological measures or when orthogonalizing physiological measures to the motion parameters, suggesting the latter did not make a major contribution to the results.

Taken together, the joint interpretation of behavioral loadings and their associated brain activation patterns points to distinct FCP that were mobilized in a coordinated manner and constituted the major “building blocks” of the various emotions elicited by movies. The robustness of these patterns across different individuals was backed up by our iterative bootstrap procedure (see Materials and Methods) and cross-validation (see Supplementary Results, Section B).

In order to further investigate the functional role of LVs identified above (Fig. 6) and examine their similarity with discrete emotion categories, as rated by participants for the same event (as in Fig. 5), we performed a correlation analysis (Pearson coefficient) to relate each discrete emotion profile to the 6 different LVs. This analysis highlighted that each of the different LVs identified by our data-driven PLSC analysis contributed to different emotions, but to variable degrees, and that they generally held meaningful relationships with discrete categorical labels (see Supplementary Results, Section B).

### Comparison with classic emotion models

Finally, we asked how our componential model compared with other approaches used in previous brain imaging studies which typically characterize emotions in terms of valence and arousal dimensions. To do so, we applied the same PLSC approach as above but now using the feeling component alone, which encompasses features corresponding to valence and arousal ratings as employed in many studies (see Table 1). Remarkably, this feeling-based analysis revealed that only 2 significant LVs emerged to account for the observed configuration of behavioral and neural data (see Supplementary Results, Section C). Behaviorally, 1 LV mainly encoded a valence dimension (with high but opposite loadings for feeling “good” and “feeling bad”) and the other encoded arousal (with high loadings on “intense” and “long-lasting” emotions). Neurally, corresponding brain saliencies were fully consistent with previous findings in the literature for the 2 classic dimensions of valence and arousal (Knutson et al. 2014; Meaux and Vuilleumier 2015), with predominant modulations of the first LV in VMPFC, amygdala, VTA (positive loadings) as well as insula, dorsal ACC, and thalamus (negative loadings), and modulations of the second LV in insula, rostral ACC, and brainstem (positive loadings). However, these 2 LVs showed much less power in explaining the shared brain–behavior covariance than our full componential model (see Supplementary Result, Section C for details).

## Discussion

Our study demonstrates a new data-driven approach to uncover the neural substrates of emotion without imposing any a priori or pre-defined categories, dimensions, or stimulus classes. Instead, our experimental design relies on a previously defined theoretical model (CPM) and established concepts of emotion in psychology (GRID features) that go beyond dimensional or categorical models. Our results reveal at least 6 distinct FCP, engaged in response to emotional experiences across a large set of naturalistic movies, and characterized as LV (1–6) linking particular combinations of emotion features with particular patterns of brain-wide activity. The 6 FCPs identified here appear to encode appraisal of value, novelty, hedonic experience, goal monitoring, caring for others, and approach tendency/curiosity. Their neuroanatomical underpinnings involve distributed brain networks, including regions consistently implicated in emotion processing (e.g. amygdala, VMPFC, insula, VTA) and also other regions (e.g. fronto-parietal areas associated with sensorimotor function, social cognition, attentional control), which accord well with appraisal theories and componential models of emotion (Scherer 2009a; Fontaine et al. 2013).

Importantly, in this framework, each FCP contributes to different emotion types independently and to different degrees; hence, 1 particular FCP can be mobilized in more than 1 emotion as illustrated by strong correlations observed between individual FCPs and specific discrete emotions. We also found that some of the FCPs have higher loadings on 1 particular component (e.g. appraisal or feeling) although most include mixed features that do not purely correspond to 1 unique component as traditionally distinguished in CPM (see Fig. 1). This may reflect a limitation of subjective ratings that fail to capture pure componential processes, or more fundamental principles of brain function whereby any cognitive or affective task emerge from the interaction of multiple neural systems.

The FCP identified with the current methodology bear several similarities with basic dimensions of “core affect” considered in past research (such as valence or arousal; Russell 2003; Fontaine et al. 2007), but also differ in important ways that offer novel insights into the componential nature of emotions. FCP-1 clearly resembles a bipolar valence dimension, often highlighted as the dominant aspect of emotional experience across many studies (Russell 2003). Here FCP-1-related bipolar elements of motivational values and feelings with brain regions implicated in affective evaluative processes (such as amygdala and VTA) and affect-based decision-making (such as VMPFC, OFC, and insula). Mobilization of this network is tightly associated with positive versus negative emotion categories, and thus consistent with valence being a major constituent of core affect (Russell and Barrett 1999). However, an involvement of action features (e.g. urge to stop) and brain areas (e.g. VMPFC) associated with motivation and decision-making, but low loading on feeling intensity, suggest that FCP-1 may relate to the theoretical construct of “wanting” (or not wanting; Berridge 2009), rather than to the hedonic experience of “liking” (Berridge 2009).

In contrast, FCP-3 is also characterized by elements of pleasantness, but with high intensity feeling features and motor expression features, suggesting a more direct implication in hedonic experience, reminiscent of the “liking” rather than “wanting” aspect of valence (Berridge 2009). Accordingly, this FCP shows strong positive correlation with joy and love, but negative correlation with sadness and anxiety, like FCP-1 (Berridge 2009). Neural activation in regions overlapping with motor face areas in operculum and preSMA would be consistent with networks controlling smiling and laughing (Caruana et al. 2015). Electrical stimulation of medial premotor cortices may elicit both laughter and mirth (Caruana et al. 2015), and covert activation of zygomatic muscles is a reliable marker of positive valence (Delplanque et al. 2009). Unfortunately, because of technical limitations, facial EMG was not collected in the MRI scanner. Our findings of 2 distinct networks (FCP-1 and FCP-3) correlating with valence features converge with growing evidence that this dimension may not have a unique psychological or neural underpinning (Kron et al. 2015; Berridge 2019). Animal research suggests that hedonic aspects of “liking” are represented in local “hotspots” of ventral striatum (accumbens and pallidum), whereas motivational aspects of “wanting” are encoded in more distributed sectors of dopamine circuits (Berridge 2009). Here we observed differential modulations of VTA and basal ganglia in both FCP-1 and FCP-3, but no distinctive activation patterns within the striatum with our current resolution and threshold. Nevertheless, predominant increases in VMPCF and amygdala for motivational features but in insula and sensorimotor operculum for pleasurable features accord well with a differential recruitment of these areas during the choice or expectation of rewards and the consumption of rewards, respectively (O’Doherty et al. 2002; Small et al. 2008).

Another core process, FCP-2, appears to selectively encode novelty, with several memory-related areas, sensory areas, and prefrontal areas. The amygdala is also engaged, in keeping with its role in novelty detection (Schwartz et al. 2003). This FCP is highly correlated with ratings of surprise and may constitute a key substrate for this non-valenced emotion. A prominence of novelty neatly accords with theories of emotion that place it as a frequent dimension after valence and arousal (Fontaine et al. 2007), or an essential appraisal initiating emotion episodes (Scherer 2009a). Our data suggest that novelty detection is not only an important constituent of emotion but also critically relies on memory functions evaluating contextual information. Notably, however, FCP-2 is low on action features and feeling intensity, suggesting it does not reflect a dimension of arousal or alertness.

The FCP-4 bore similarities with FCP-2 but with higher loadings on appraisals of goals, expectations, and attention, which covaries with activation of brain networks mediating social cognition and attentional reorienting mechanisms (Corbetta and Shulman 2002). It also correlates with ratings of surprise and anger. This pattern may reflect more complex responses to goal interruption or shifting, based on representations of intentions and action-outcome expectations. This accords with a previous fMRI study using verbal scenarios (Skerry and Saxe 2015) where appraisal features such as goal consistency, intention, or agency accounted for discriminative neural signatures in theory-of-mind networks, better than valence or arousal dimensions. More generally, these data further support appraisal theories according to which goal conduciveness and goal obstruction are key elements of emotion processing (Scherer 2009a).

FCP-5 is remarkable as it involves appraisal of goals (for someone else) with a striking mixture of high intensity feelings, no clear valence polarity, and prominent physiology features, particularly lump in the throat and warmth. This constellation is reminiscent of the experience of “being moved,” an emotional response whose nature and even existence is debated in the psychology literature (Zickfeld et al. 2019). Recent conceptual analysis in philosophy (Cova and Deonna 2014), however, argued that the state of “being moved” may hold all necessary criteria to be considered a “basic” emotion, similar to traditional kinds such as fear, anger, or happiness (Ekman 1999). Moreover, FCP-5 correlates with 2 discrete emotions of opposite valence, love and sadness, in agreement with situations typically associated with reports of “being moved” (e.g. attending a concert given by one’s child or goodbye to a soldier leaving for war). To our knowledge, this emotion has never been studied in neuroscience. Here, we find not only that elements of this experience emerged as a specific FCP, but also, they distinctively mobilized midline brain areas overlapping with the DMN. Intriguingly, DMN activity is associated with introspective processing as well as access to self-related, affectively relevant information in memory (D’Argembeau and Van der Linden 2004) converging with the claim that “being moved” may reflect the activation of core self-values (Cova and Deonna 2014) and our interpretation that FCP-5 may encode social concern or “caring for others” (Helm 2010).

Finally, FCP-6 has marginal significance, but shows a distinct pattern with appraisal features related to antisocial information and motivational features of active approach, attack, and attention, positively correlated with discrete ratings of fear and disgust. A bipolar activation map with increase in VTA, VMPFC, and sensorimotor areas, but decrease in anterior insula and rostral ACC, could putatively fit well with opponent processes of approach/curiosity versus avoidance/aversion, another common dimension in classic emotion theories (Sander et al. 2005).

Notably, the reliability of these factors across individuals was verified via a bootstrap approach in which different subsets of subjects (two-thirds of our sample) were randomly selected at every iteration of resampling, allowing us to test for the robustness/reproducibility of the LVs across a different group of subjects (remaining third of our sample). These bootstrap results demonstrated a very small variation for each factor, which implies a good reliability of the different LVs and strong generalizability across subjects. Therefore, despite natural variability in affective experience across people, our results suggest that the 6 different FCPs identified here were consistently present in different subsets of participants.

While our study provides several novel insights concerning the functional componential organization of emotions and their neural underpinnings, built on well-established theorizing (Scherer 2009a), it is not without limitations. As our approach to define LVs (in both feature and brain space) is purely data driven, our interpretation of FCPs necessarily implies post hoc speculations based on extant knowledge and previous studies. This is not without a risk of reverse inferences (Poldrack 2006), although here brain activation patterns were confronted with pre-defined behavioral features and concepts derived from precise theoretical hypothesis in psychology (Fontaine et al. 2013). Moreover, our findings appear consistent with other data-driven meta-analysis (Kober et al. 2008; Lindquist et al. 2012; Wager et al. 2015), but go beyond usual MVPA-based approaches with pre-defined categories or dimensions that do not readily account for the rich variety but also similarity between emotions. The current data therefore accord with theoretical accounts that argue against a modular and fixed organization of emotions but emphasize the role of interactive appraisal mechanisms (Scherer 2001; Sander et al. 2005) and contextual factors in the psychological construction of an emotional experience (Barrett 2017b). However, our work extends this framework by delineating specific functional components and corresponding brain networks engaged across a range of emotion-eliciting situations, rather than linking emotions to general conceptual categorization processes operating on interoceptive and exteroceptive information that generate “core affect” signals. Our study also goes beyond previous neuroimaging work that provided support to multi-componential organization of emotion networks based on post hoc meta-analysis (Kober et al. 2008; Wager et al. 2015), by designing our experimental paradigm and analysis based on a set of pre-defined, theoretically driven descriptors of specific components (including several appraisal indices).

Another potential limitation of the current study is that our 6 FCPs were derived from a limited data set (containing 40 movies) that may not embrace the whole emotion spectrum. In any case, to the best of our knowledge, our stimulus set is still much larger than any other standard neuroimaging studies in this field. Furthermore, we cover a wide range of features previously validated across a variety of emotional responses (Fontaine et al. 2013), and surmise that similar FCPs would occur in other emotions not elicited here (perhaps combined with additional networks).

In addition, emotions elicited by movies might most often be vicarious, in that events are not directly happening to the viewer in the real world, which may limit the generalization of our results to first-person emotions and highlights the need for using more immersive paradigms in future research to elicit first-person emotions (e.g. using games or virtual reality). However, such limitations appear even more severe in past research using pictorial stimuli such as faces or scenes. Naturalistic elicitation of emotions also implied that participants were not self-monitoring and it is therefore hard to measure their level of attendance to each video clip. However, during debriefing, all participants admitted that they found all clips engaging and online monitoring through eyetracker recordings ensured they stayed awake with eyes open and looking at the screen. Also, we used a single HRF function for every emotional event in movie clips to model the BOLD signal, which is a standard practice for fMRI studies but comes with potential limitations such as neglecting carry-over, habituation, or sensitization effects. However, this should apply equally to all emotion features and would limit our analysis sensitivity rather than create spurious results. Moreover, our analyses were performed over several hours of experiment from 20 participants and the total amount of both neural and behavioral data is larger longer than most precious fMRI studies. In spite of this and statistical tests confirming the reliability of results, it would be insightful to run similar analyses over a larger pool of participants and/or in a different context. Running similar studies with higher temporal resolution could also help to shed light on potential causalities in these processes.

We also note that we found no component uniquely associated with peripheral physiology changes during emotion events. In our fMRI analysis, we chose not to include physiology parameters as nuisance regressors but only standard head motion-related confounds, in order to maximize our sensitivity for detecting concomitant brain patterns. However, this might also increase the risk of spurious neural signals, although we presume this is unlikely given the lack of consistent loading associated with RR or HR in our analysis, and the lack of any systematic correlation between head motion and physiology parameters (see Materials and Methods). Nevertheless, there might be some aspects of noise stemmed from HR (because of its actual higher frequency than the fMRI images) that may not have been accounted for, therefore additional research is required to pinpoint brain processes governing peripheral physiology effects while carefully ruling out noise confounds in fMRI signals. Moreover, previous work (Menétrey et al. 2022) suggested that peripheral physiology changes induced by emotion can be reliably predicted by other GRID components, which might explain that physiology indices showed no distinctive patterns overall in the current study.

Lastly, a striking aspect of our results is the absence of a FCP linking behavioral features and brain networks corresponding to the construct of arousal, despite this being the second major ingredient of “core affect” in classic theories of emotions (Russell and Barrett 1999). This might stem from insensitive features related to arousal and limited measures of physiological responses, but this seems unlikely given our control analysis based in feeling items that revealed 2 LVs consistent with valence and arousal (at both the behavioral and neural levels). Alternatively, our findings may support the notion that arousal does not constitute a single well-defined functional process, but encompasses variable aspects of affect, vigilance, and autonomous nervous functions (Satpute et al. 2019). Arousal may therefore emerge as a single dimension only when using subjective affect ratings and ignoring more comprehensive appraisal components. Moreover, our control analysis based on feeling items was found to account for less variance in the data than our full componential model, even though it could neatly replicate previous work on valence and arousal, indicating that other component features provide additional and discriminative information to characterize the underlying functional organization of emotions across various situations.

In sum, our study offers a new approach to study human emotions using both theory-based and empirically validated parameters without pre-defined categories or dimensions. Our results provide new insights into the functional structure of affective processes and their relation to particular brain substrates, adding support to componential models (such as the CPM or constructionist framework) and shedding light on neglected emotions such as “being moved.” In doing so, our work goes some way toward elucidating the constitutive ingredients of emotions and linking them to network accounts of brain function, using data-driven methodology. Nevertheless, we acknowledge that further studies are necessary to verify these findings, confirm the replicability in different contexts, and determine any causality among components.

## Supplementary Material

FInalSubmission_Supplementary_Information_bhad093

## Data Availability

All data underlying the results reported in this manuscript are available upon request by qualified researchers for their own use (noncommercial) from the following link: https://doi.org/10.26037/yareta:bws2cxneqvfrppxy4mf44gueyi.

## CRediT authors statement

Gelareh Mohammadi (Conceptualization, Data curation, Formal analysis, Investigation, Methodology, Project administration, Validation, Visualization, Writing – original draft, Writing – review – editing), Dimitri Van de Ville (Methodology, Supervision, Writing – review – editing), Patrik Vuilleumier (Conceptualization, Funding acquisition, Methodology, Supervision, Writing – review – editing)

## Code availability

The custom MATLAB codes used to analyze the data that support the results reported in this manuscript are available from the corresponding authors for noncommercial use only.
