# Supplementary material for: Brain networks subserving functional core processes of emotions identified with componential modeling
Source: Cereb Cortex. 2023 Mar 22;33(12):7993–8010. doi: 10.1093/cercor/bhad093 (PMC10267645; doi:10.1093/cercor/bhad093)
Supplement: FInalSubmission_Supplementary_Information_bhad093 [file finalsubmission_supplementary_information_bhad093.pdf]

Supplementary information

Supplementary Methods:

A. CoreGRID descriptor selection

The CoreGRID instrument includes 63 semantic concepts capturing descriptors relevant to the five major component in the component model[1]. Several concepts in this instrument postulate a first-person perspective (where the participant is the subject of an action or is actively involved in a real situation with potential implications to oneself) and were therefore not applicable to our study where the participant is observing events in a movie. Some example of such descriptors is as follows:

- "… important and relevant for my goals"
- "… caused by my own behaviour"
- "… I spoke slower/faster"
- "… I wanted to handover the initiatives to someone else"
- "… I had resources to avoid or modify consequences"

Hence all such descriptors were eliminated from the list, which resulted in 39 items to be used for ratings (see Supplementary Table 1 for the list of descriptors in the preliminary stimuli selection study). In addition, the item "speech disturbances" was also eliminated for the fMRI study as participants are restricted in moving or talking inside the scanner. Moreover, for 4 physiology descriptors including "heartbeat getting faster", "breathing slowing down", "felt warm", and "sweat", and 2 facial expressions of "smiling" and "frowning", we decided to use objective measures through sensors, and therefore these 6 descriptors were included in the preliminary selection study but excluded from subjective ratings in the fMRI study. Thus, in the final experiment, participants had to judge their affective experience in terms of the remaining 32 descriptors from the GRID.

Supplementary Table 1: Selected GRID descriptors. This table shows the list of GRID descriptors selected for the preliminary stimulus selection study. For the final fMRI study, 38 items were retained out of which 6 were collected objectively through sensors (but due to technical reasons, only HR and RR could be used in final analysis, see main text).

	**GRID selected descriptors**	*Component*
	While watching this movie, did you...	
1	think it was incongruent with your standards/ideas?	Appraisal
2	feel that the event was unpredictable?	Appraisal
3	feel the event occurred suddenly?	Appraisal
4	think the event was caused by chance?	Appraisal
5	think that the consequence was predictable?	Appraisal
6	feel it was unpleasant for someone else?	Appraisal
7	think it was important for somebody's goal or need?	Appraisal
8	think it violated laws/social norms?	Appraisal
9	feel in itself was unpleasant for you?	Motivation
10	want the situation to continue?	Motivation
11	feel the urge to stop what was happening?	Motivation
12	want to undo what was happening?	Motivation
13	lack the motivated to pay attention to the scene?	Motivation
14	want to destroy s.th.?	Motivation
15	want to damage, hit or say s.th. that hurts?	Motivation
16	want to tackle the situation and do s.th.?	Motivation
17	have a feeling of lump in the throat?	Physiology
18	have stomach trouble?	Physiology
19	experience muscles tensing?	Physiology
20	feel warm?	Physiology
21	sweat?	Physiology
22	feel heartbeat getting faster?	Physiology
23	feel breathing getting faster?	Physiology
24	feel breathing slowing down?	Physiology
25	produce abrupt body movement?	Expression
26	close your eyes?	Expression
27	press lips together?	Expression
28	have the jaw drop?	Expression
29	show tears?	Expression
30	have eyebrow go up?	Expression
31	smile?	Expression
32	frown?	Expression
33	produce speech disturbances?	Feeling
34	feel good?	Feeling
35	feel bad?	Feeling
36	feel calm?	Feeling
37	feel strong?	Feeling
38	feel an intense emotional state?	Feeling
39	experience an emotional state for a long time?	Feeling

B. Emotional segment selection

Although the initial assessment to select video clips was done on the basis of global judgments, to avoid any confound of multiple events related to different appraisal components within a clip, we analysed each single emotional event separately for the final fMRI study. The selection of emotional events was semi-manual and done through a separate experiment. In that experiment, five participants were asked to watch the video clips and continuously rate the emotional content using the CARMA tool (software for Continuous Affect Rating and Media Annotation)[2]. The ratings were quantified on a range from 0 to 100 and, for each participant, ratings above the mean value 32 (the value has been rounded) were selected as emotional moments. Events that were annotated as emotional by a majority of participants (3 or more participants) were selected as emotional segments. The start and end of each segment was manually adjusted to have the same length across all participants in the final fRMI study. Supplementary Table 2 lists the name of the films for each clip and the number of emotional events selected in that clip.

This selection of emotional events resulted in a non-uniform distribution of discrete emotions, as depicted in Figure 3a. This is unlike the global judgments made on the initial video clip dataset and used to select the final experimental movie list, indicating that such global assessment of video clips (as done in may studies) may not necessarily apply to all its single events within the clip, and thus highlighting the importance of focusing on short segments to evaluate felt emotions. Please note that this non-uniform distribution might be considered as one limitation of the current study but does not necessarily affect the main results since our main goal is to cover a wide range of componential space, not to elicit or compare specific discrete emotions.

Supplementary Table 2: High saliency brain areas. This table lists the name of the films for each clip, its duration, the original emotion label as collected in another study and number of events selected in each clip.

Films	Duration (s)	Related Emotion	Segments
12 Years A Slave	120	Anger	4
28 Days Later	143	Fear	4
28 Days Later	49	Anxiety	4
28 Days Later	125	Fear	4
A Fish Called Wanda	173	Joy	4
American History X	77	Anxiety	4
Bambi	86	Love	2
Batman Returns	73	Surprise	3
Cry Freedom	132	Disgust	4
Dangerous Minds	128	Sadness	2
Edward Scissorhands	93	Calm	4
Forrest Gump	121	Love	3
Ghost	215	Love	4
Hotel Rwanda	209	Sadness	4
Hotel Rwanda	96	Sadness	2
Hotel Rwanda	97	Anger	3
Kill Bill 1	68	Surprise	2
Kill Bill 1	144	Surprise	2
Life Is Beautiful	127	Anger	3
Life Is Beautiful	105	Love	2
Love Actually	76	Joy	3
Love Actually	104	Joy	4
Love Actually	28	Satisfaction	1
Mr. Bean's Holiday	128	Joy	4
My Girl	146	Sadness	2
Planet Earth	66	Calm	3
Pride And Prejudice	91	Calm	3
Remember The Titans	132	Satisfaction	3
Schindler's List	115	Disgust	2
Scream 2	215	Fear	4
Terminator 2	131	Satisfaction	4
The Dentist	56	Fear	2
The Fly	81	Anxiety	3
The Pianist	81	Anger	2
The Pianist	117	Anxiety	2
There Is Something About Mary	175	Surprise	4
The Shining	85	Satisfaction	2
Trainspotting	104	Disgust	3
Trainspotting	62	Disgust	1
Wild Alaska	62	Calm	2

C. fMRI session

Each fMRI session started off by giving instructions regarding the experiment and fMRI acquisition protocol. Next, participants had to fill in the required forms and completed a 16-item Brief Mood Introspection Scale (BMIS) mood questionnaire[3]. When the participant was ready, (s)he entered the scanner and the physiology collecting devices, EMG, headphones, and eye-tracker were set up. After checking all physiology signals and calibrating the eye-tracker, the actual acquisition started. Each video clip was played during one single run and

each run lasted ~164s including a short initial preparation time and final washout clip. There was an interval of ~30 s between consecutive runs. Supplementary Figure 1 illustrates the sequence of events inside the fMRI scanner. On average, the overall time inside the scanner for each session was about 32 minutes, excluding the setup time, calibration and structural MRI acquisition.

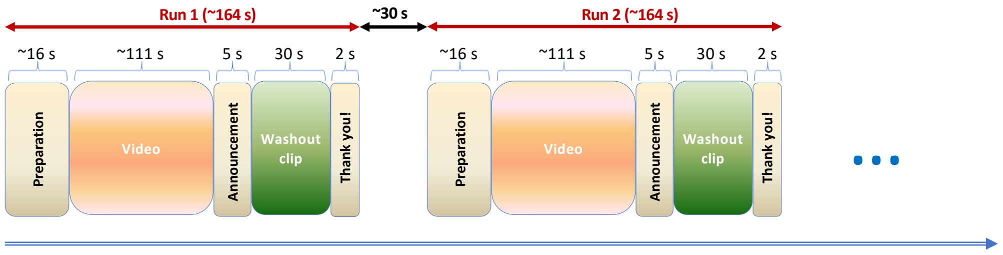

Supplementary Figure 1: fMRI experiment design. This figure shows the design of experiment when participants where inside the scanner and the average time course of every phase. There was about 30 seconds intervals between two consecutive runs.

D. Behavioral session:

Similar to fMRI session, at the beginning of each behavioral session, participants were briefed on the procedure to evaluate their felt emotions. The experiment started by asking them to answer a 10-item Big Five Inventory (BFI) personality questionnaire[4], followed by playing the same video clips as those seen in the fMRI session. Emotional segments were highlighted by a red frame to notify the participant of the video segment/event, (s)he had to assess. After each emotional segment, the video was paused, and the participant had to answer 32 questions covering the GRID component items and 2 questions regarding the two most dominant discrete emotions they felt. Upon completion of the assessment, video resumed from where paused. Each behavioral session took on average about 110 minutes excluding the instruction time. Supplementary Figure 2 illustrates the behavioral rating experiment.

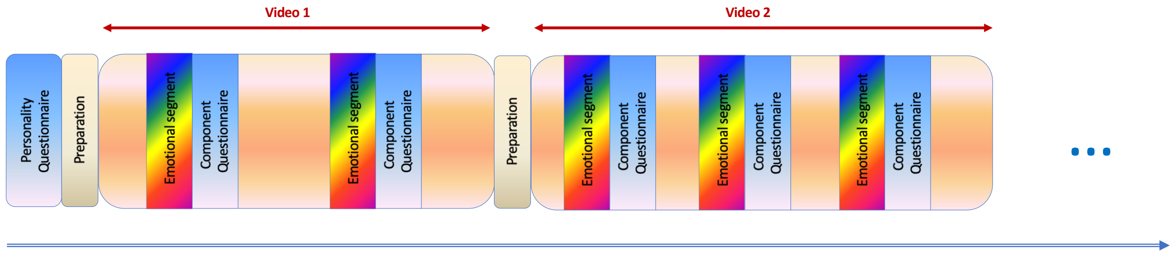

Supplementary Figure 2: Behavioral experiment design. This figure shows the design of experiment when participants were outside fMRI scanner and assessed their subjective emotion experience for every highlighted emotional segment in movies.

Supplementary Table 3: High saliency brain areas. This table lists the brain areas (excluding brain stem) as defined in AAL atlas with high average saliencies (>3 or <-3) corresponding to each of the 6 latent variables. Average values across voxels of a given region may artificially increase or decrease the apparent importance of some regions relative to others.

Region	LV1	LV2	LV3	LV4	LV5	LV6
'Precentral_L'	-	3.52	3.27	-	-	-
'Precentral_R'	-	-	3.46	-3.38	-	-
'Frontal_Sup_L'	-	-	-	-	-	3.14
'Frontal_Sup_R'	-	-	-	-	-	3.32
'Frontal_Sup_Orb_L'	-	-	-	-3.37	-	-
'Frontal_Sup_Orb_R'	3.25	3.34	-3.24	-	-	-
'Frontal_Mid_L'	-	3.17	-	-3.11	3.42	-
'Frontal_Mid_R'	-	-	-	-3.28	-	-
'Frontal_Mid_Orb_L'	3.60	-	-	-	-	-
'Frontal_Mid_Orb_R'	3.26	-	-	-3.15	-	-
'Frontal_Inf_Oper_L'	-3.39	3.49	-	-	-	-3.11
'Frontal_Inf_Oper_R'	-3.28	-	3.18	-	-	-3.16
'Frontal_Inf_Tri_L'	-	3.26	-	-	3.38	-
'Frontal_Inf_Tri_R'	-	3.22	-	-	-	-
'Frontal_Inf_Orb_R'	-	-	-	-	3.10	-3.31
'Rolandic_Oper_L'	-3.47	-	3.77	-	-	-
'Rolandic_Oper_R'	-3.25	-	4.10	-	-	-
'Supp_Motor_Area_L'	-	3.16	3.28	-	-	-
'Supp_Motor_Area_R'	-	3.14	3.17	-3.55	-	-
'Olfactory_L'	-	-	-	-	3.46	-
'Olfactory_R'	-	-	-	-	3.44	-
'Frontal_Sup_Medial_L'	-	3.42	-	-	3.16	-
'Frontal_Sup_Medial_R'	-	3.48	-	-	-	-
'Frontal_Med_Orb_L'	-	-	-	-3.47	3.26	3.27
'Frontal_Med_Orb_R'	-	3.22	-3.14	-3.36	3.57	-
'Rectus_L'	3.74	-	-	-3.49	-	-
'Rectus_R'	3.49	3.43	-3.33	-3.43	3.34	-
'Insula_L'	-3.88	-	3.53	-	3.39	-3.81
'Insula_R'	-3.79	-	3.58	-	-	-3.64
'Cingulum_Ant_L'	-	-	-	-3.31	3.72	-
'Cingulum_Ant_R'	-3.16	-	-	-3.20	3.69	-
'Cingulum_Mid_L'	-3.35	-	3.45	-3.14	3.20	-
'Cingulum_Mid_R'	-3.37	-	-	-3.32	3.32	-
'Cingulum_Post_L'	-	-	-	-	3.28	3.67
'Cingulum_Post_R'	-3.21	-	-4.23	-	3.55	-
'Hippocampus_L'	-	-	-	-	-	3.42
'Hippocampus_R'	-3.57	-	-	-	-	3.26
'ParaHippocampal_L'	-	-	-	-	3.15	-
'Amygdala_L'	-3.19	-	-	-	-	-
'Amygdala_R'	-	-	-	-	-	3.36
'Calcarine_L'	-3.24	3.11	-	-	-	-
'Cuneus_L'	-3.14	-	-	-	-	-
'Cuneus_R'	-3.61	-	-	-	-	-
'Lingual_L'	-3.43	-	-	-	-	-
'Lingual_R'	-3.22	-	-	-	-	-
'Occipital_Sup_L'	-3.40	-	-	-3.36	-	-
'Occipital_Sup_R'	-	-	-	-3.73	-3.18	-
'Occipital_Mid_L'	-3.96	-	-	-	-	-
'Occipital_Mid_R'	-3.80	-	-	-	-3.18	-
'Occipital_Inf_L'	-3.55	-	-	-	-	-
'Occipital_Inf_R'	-	-	-3.16	-	-	-

'Fusiform_L'	-3.34	-	-	-	-	-
'Fusiform_R'	-3.23	-	-	-	-	-
'Postcentral_L'	-	-	3.67	-3.18	-	3.15
'Postcentral_R'	-	-	3.69	-3.67	-	3.43
'Parietal_Sup_L'	-3.29	-	-	-3.28	-	-
'Parietal_Sup_R'	-3.44	-	-	-3.85	-	3.18
'Parietal_Inf_L'	-3.43	-	-	-3.39	-	-
'Parietal_Inf_R'	-3.35	-	-	-4.36	-	-
'SupraMarginal_L'	-4.87	3.24	3.15	-	3.72	-
'SupraMarginal_R'	-4.55	-	-	-	-	-
'Angular_L'	-	-	-	-	4.43	-
'Angular_R'	-	-	-3.38	-3.74	3.21	-
'Precuneus_L'	-3.65	3.17	-	-	-	3.17
'Precuneus_R'	-3.61	-	-3.69	-	-	3.17
'Paracentral_Lobule_L'	-	-	3.42	-3.60	-	3.52
'Paracentral_Lobule_R'	-	-	3.29	-3.52	-	3.12
'Caudate_L'	-3.17	-	-	-	-	-
'Caudate_R'	-3.38	-	-	-	-	3.32
'Putamen_L'	-3.37	-	-	-	3.20	-
'Putamen_R'	-3.52	-	3.33	-	3.14	-
'Pallidum_R'	-3.52	-	-	3.39	3.20	-
'Thalamus_L'	-4.08	-	-	-	-	-
'Thalamus_R'	-4.20	-	-	-	-	-
'Heschl_L'	-	-	3.37	-	-	-
'Heschl_R'	-	3.30	3.49	-	-	-
'Temporal_Sup_L'	-	3.57	3.44	-	-	-
'Temporal_Sup_R'	-	3.81	-	3.11	-	-
'Temporal_Pole_Sup_L'	3.75	3.34	3.90	-	3.20	-3.18
'Temporal_Pole_Sup_R'	3.13	3.25	3.70	-	-	-
'Temporal_Mid_L'	-	3.52	-	-	-	-
'Temporal_Mid_R'	-3.64	3.87	-3.52	-	-	-
'Temporal_Pole_Mid_L'	-	-	-	-	3.31	-
'Temporal_Pole_Mid_R'	-	-	-3.13	-	-	-
'Temporal_Inf_L'	-	3.18	-	-	-	-
'Temporal_Inf_R'	-3.55	-	-	-4.04	-	-

Supplementary Results

A. Brain Saliencies:

The partial least square correlation method estimates saliency values for each voxel in the brain. To summarise the list of regions with the highest saliencies, we used Automated Anatomical Labelling (AAL) atlas[5] and reported the results in Supplementary Table 3. The reported values are the average saliencies across all voxels of a given area in the atlas (with cut-off point = 3 for positive values and -3 for negative values) in order to consider only the strongest and largest cluster and facilitate comparison with previous studies. The absolute values might artificially reduce or augment the apparent importance of some regions

relative to others due to differences in region size in the atlas, but provide a quantitative index reflecting the overall contribution of each AAL region to each latent variable. For clarity, other regions with lower saliencies (surviving cut-off points of 2.5/-2.5) are not listed in order to exclude smaller effects and reduce blurring caused by fuzzy borders between neighbouring regions.

B. Cross validation control

Bootstrap is the main approach in the literature to evaluate the generalisability and reliability of PLSC as an unsupervised statistical method, as we also used here. Nevertheless, to further evaluate the reliability of our method and result using another complementary perspective (similar to traditional approaches with other multivariate techniques), we also performed a K-fold cross validation with 5 folds to examine how well our PLSC results could be reproduced across different individuals. At every iteration of cross-validation, the recordings of 4 participants out of 20 were left out as the test set, and data from the remaining 16 participants were used to train the PLSC and estimate the corresponding brain and behaviour loadings. Similar to bootstrap analysis, in order to align the order of latent variables obtained at every iteration, Procrustes rotation was applied. Then the loadings obtained from the training set were applied to the unseen data in the test set in order to compute the brain scores in the latent space. Finally, to compare the results from K-fold and original methods, we computed the correlation between the brain scores from the K-fold results and those from the original PLSC, and we depicted this correlation for each latent variable (LV) in a scatter plot of the scores from the two different methods. Supplementary Figure 3 shows the scatter plot and the correlation values obtained for the 6 significant LVs identified in our original analysis. As evident from the result, the scores (and so the loadings) obtained for brain activations and behaviour ratings in K-fold cross-validation are very similar to the scores from the original PLSC. As expected, the correlation coefficients between the original result and k-fold result are the highest for the first LVs (>.95 for LV1 to LV4) and then decrease for loadings with less significance (LV5 and LV6). This result converges with our bootstrap to confirm, once more, the reliability of the PLSC approach and the generalisability of the results across individuals when only using a portion of the sample to train our model and test it on the remaining sample.

C. Comparison with classic emotion models

In order to compare our results obtained with our componential model with traditional accounts of emotions that mostly consider subjective experiential features (e.g. pleasantness or unpleasantness of particular stimuli or situations), we performed a similar PLSC analysis on the feeling component items which mainly describe valence and arousal features similar to classic bidimensional models (see Table 1 for the list of questions). Consistent with the previous studies in the field, this control PLSC analysis resulted in only two significant latent variables (LVs), one consistent with valence and the other consistent with arousal. These findings demonstrate that these two dimensions can be retrieved in a data-driven method but only accounting for feeling component.

The corresponding behavioural loadings are shown in Supplementary Figure S4, where the first latent variable (LV1) is mostly sensitive to ratings of feeling good, feeling bad, or feeling calm that can be interpreted as valence, while the second latent variable (LV2) is sensitive to feelings of long emotional states and feeling of intense emotions that suggest an arousal dimension. Supplementary Figure S5 highlights the brain saliency maps corresponding to these two LVs.

The first dominant latent variable (LV1) encoding valence exhibited the most significant positive weights in VMPFC, VTA, amygdala, and auditory cortex, and the most significant negative weights in anterior insula, dorsal ACC, lateral occipital cortex, thalamus, and putamen. The second latent variable (LV2) encoding arousal showed the most significant positive weights in rostral ACC, dorsal insula, SMA/Pre-SMA, lower brainstem (pons), superior parietal cortex, as well as IFG and frontal operculum. Conversely, negative weights were observed in STS from posterior to anterior sections, MPFC, lateral occipital, and medial parietal areas. These networks are generally consistent with the finding of previous brain imaging studies on valence and arousal[6,7].

In addition, to compare the explained covariance from the full component model relative to this control analysis using only items from the feeling component, we also controlled for differences in the dimensionality of the models (e.g. two PLSCs with same number of dimensions) and performed another PLSC with the feeling features alone but now including the same number of possible dimensions in affective space.

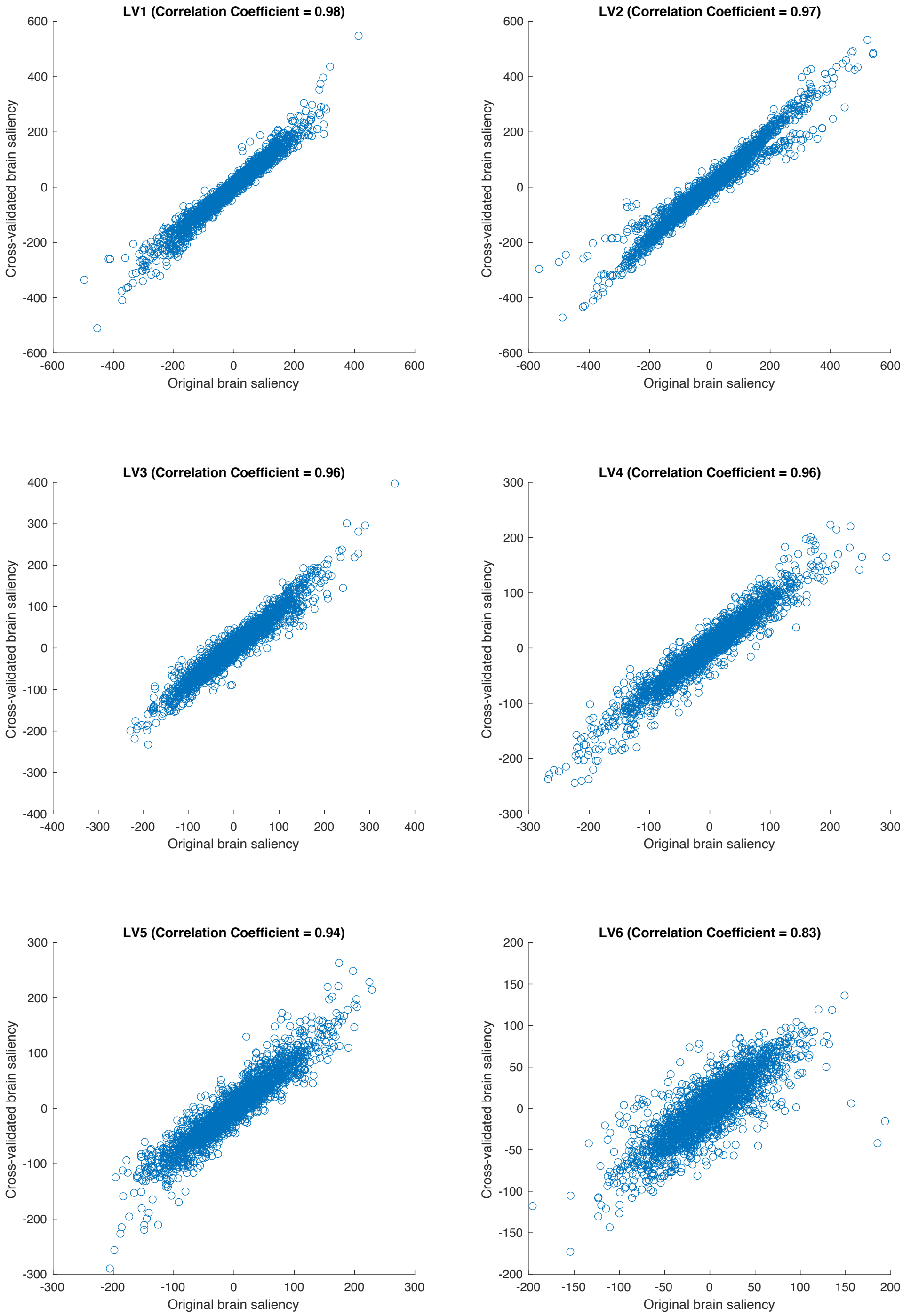

Supplementary Figure 3: Cross-validation scatter plots. Each scatter plot corresponds to the brain activity scores obtained from one of the significant latent variables. The X-axis indicates the brain scores from the original model with all participants, and the Y-axis indicates the brain scores obtained using the cross-validation approach with a subset of participants.

Specifically, we used all 34 behaviorial GRID features where all the items except those from the feeling component were randomly permuted to ensure their information is scrambled. This method allowed us to guarantee that results from the original PLSC could be comparable

with the results from the feeling-based analysis and examine if a bidimensional model (valence x arousal) was sufficient to account for our data, and at the same time rule out that differences between these models could simply be due to their dimensionality. This second control analysis again produced only two significant latent variables where the first one mainly loaded on feeling items related to valence ($p<0.0001$, 11.5%) and the second loaded on the feeling items corresponding to arousal ($p<0.0001$, 7.1%), whereas all other GRID features produced null or non-discriminative loadings (see Supplementary Figure 6). The corresponding brain saliency maps for these two LVs showed virtually identical activation patterns as found with the first control analysis and illustrated in Supplementary Figure 5. Importantly, comparing the explained covariance from the different models indicates that the model with the full components (original PLSC with 6 LVs) captures about 49% of the brain-behavior covariance in our data, while this reduces to about 19% when considering only feeling items and eliminating the information from other components, a difference that cannot be accounted for by the dimensionality of models.

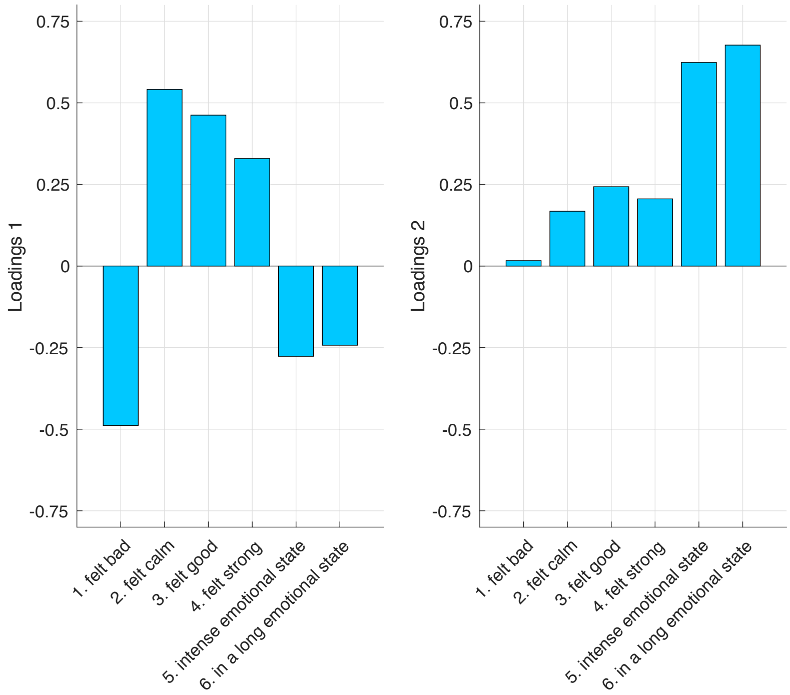

Supplementary Figure 4: Loadings of partial least square correlation in first control analysis. Loadings of PLSC for Feeling items corresponding to the two significant latent variables (1-2) identified in this analysis, respectively interpreted as valence and arousal. Each latent variable with its loading vector corresponds to a distinctive brain activity map that is shown in Supplementary Figure 5.

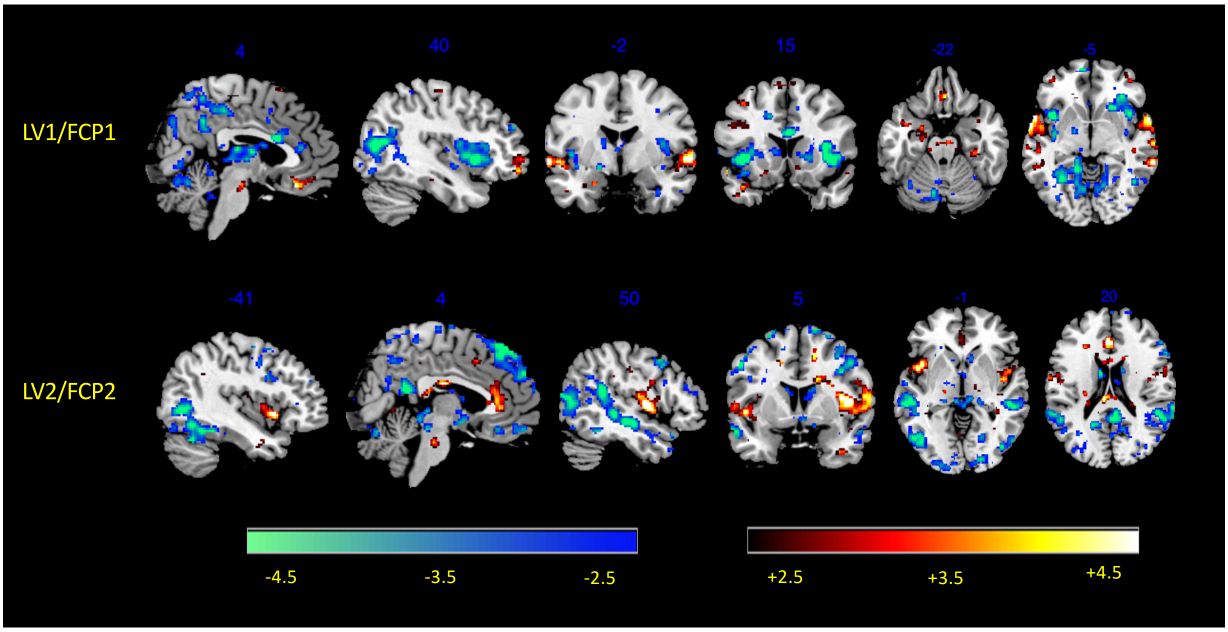

Supplementary Figure 5: Brain saliency maps in control analysis. Brain activity maps of relative saliencies corresponding to the two significant functional core processes, a.k.a. latent variables (LV), obtained by a PLS analysis using only feeling-component ratings. The red spectrum accounts for positive saliencies above +2.5 and blue spectrum corresponds to negative saliencies below -2.5.

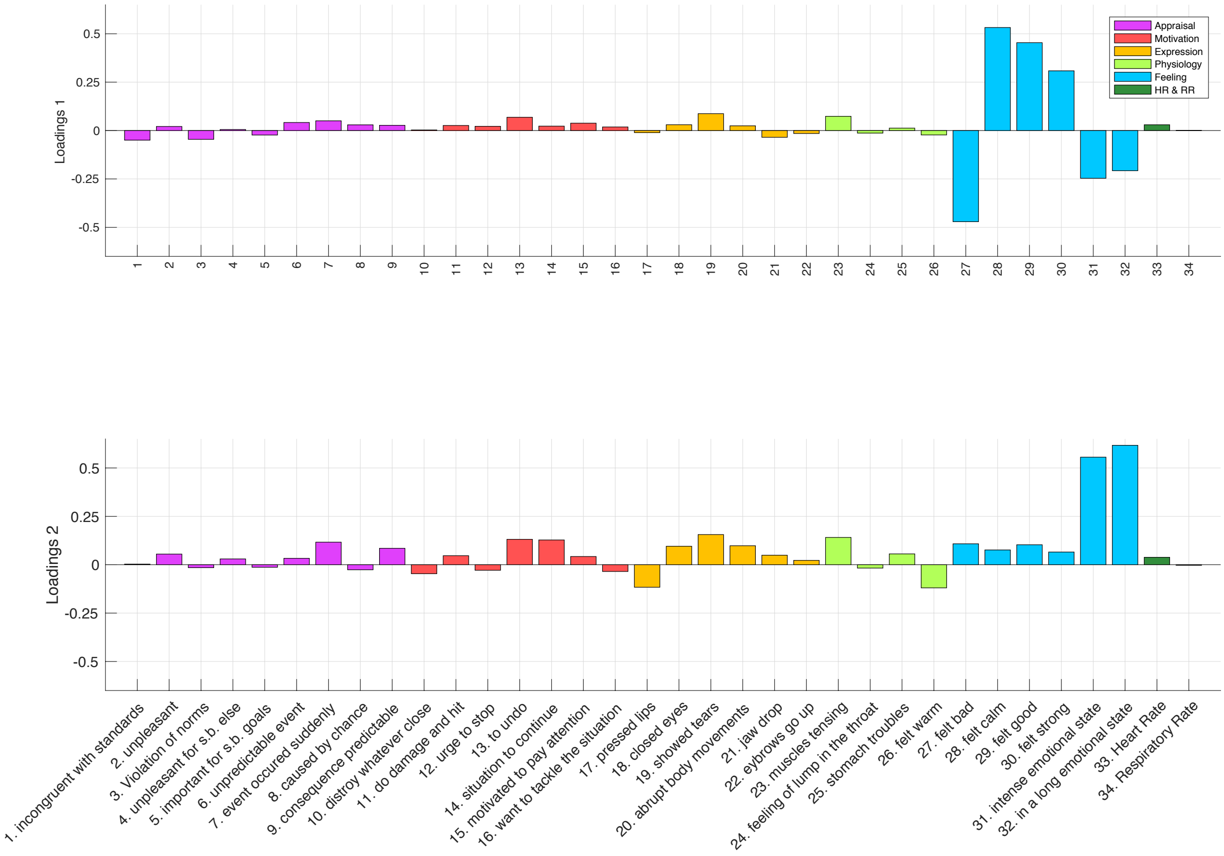

Supplementary Figure 6: Loadings of partial least square correlation in our second control analysis. Loadings of PLSC when all GRID items except those from the feeling component were permuted. Two significant latent variables (1-2) emerged, respectively interpreted as valence and arousal. This analysis was done to compare the explained covariance of full component model with an alternative feeling-based model comparable to studies based on bidimensional theories of emotions.

D. Discrete emotion and latent variables correlation:

To examine the similarity between each latent variable and the discrete emotion profiles, a Pearson correlation analysis was performed. Supplementary Figure 3 depicts the significance of these correlations, representing the relative implication of each LV in discrete emotion categories.

Almost all discrete emotions showed high positive or negative correlations with the first latent variable LV1 (encoding appraisals of values or valence), except for sadness and surprise that showed weaker or no significant correlation. The second latent variable LV2 (attributed to novelty) showed a strong positive correlation with surprise, but negative correlation with sadness and anger, while LV3 (interpreted as hedonic impact) was positively correlated with joy, satisfaction, love and calm, but negatively correlated with anxiety and sadness. LV4 (related to goals and intentions) was positively associated with surprise and negatively with anger. Interestingly, the fifth latent variable LV5 was found to be significantly correlated with sadness followed by high non-significant correlation with love, two discrete emotions of opposite valence (but consistent with a dimension of caring for others and social concern). And finally, the last latent variable LV6 (encoding dimensions of curiosity and active approach vs avoidance) showed significant correlation with ratings of fear, followed by high non-significant correlations with anxiety and disgust (associated with the negative loadings on this dimension).. Taken together, these findings highlight that each of the different LVs identified by our data driven PLSC analysis contributed to different emotions, but to variable degrees, and also that they generally held meaningful relationships with discrete categorical labels. Importantly, however, single LVs cannot be reduced to particular emotion categories or unique orthogonal dimensions such as valence or arousal.

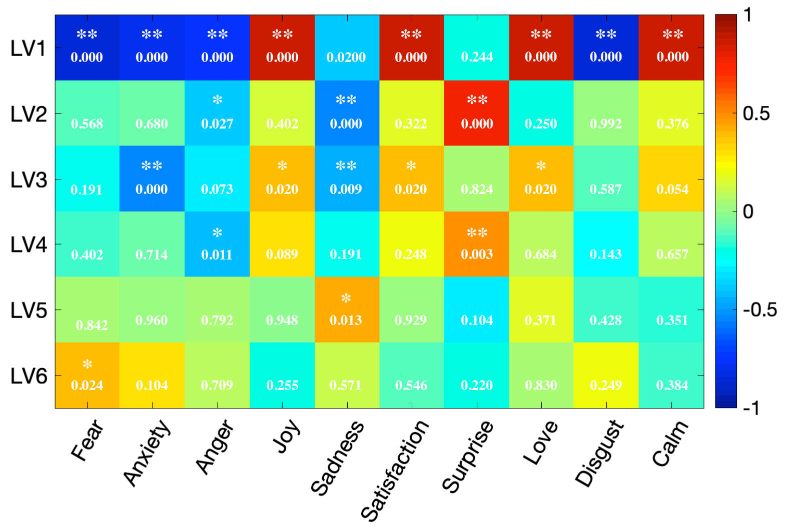

Supplementary Figure 7: Correlation analysis. Pearson correlation coefficient between latent variables (LV) and discrete emotion categories based on individual ratings of movies (** corresponds to p-value<.01 and * corresponds to p-value<.05).

Supplementary references:

1. Fontaine, J., Scherer, K. & Soriano, C. *Components of Emotional Meaning: A sourcebook*. (Oxford University Press, 2013).
2. Girard, J. M. CARMA: Software for continuous affect rating and media annotation. *J. Open Res. Softw.* **2**, (2014).
3. Mayer, J. & Gaschke, Y. The Brief Mood Introspection Scale (BMIS). *UNH Personal. Lab* (1988).
4. Anderson, C. L. *et al.* Measuring personality in one minute or less: A 10-item short version of the Big Five Inventory in English and German. *MIS Q.* **41**, 273–284 (2010).
5. Tzourio-Mazoyer, N. *et al.* Automated anatomical labeling of activations in SPM using a macroscopic anatomical parcellation of the MNI MRI single-subject brain. *Neuroimage* **15**, 273–289 (2002).
6. Wager, T. D. *et al.* A Bayesian Model of Category-Specific Emotional Brain Responses. *PLoS Comput. Biol.* **11**, 1–27 (2015).
7. Meaux, E. & Vuilleumier, P. Emotion Perception and Elicitation. in *Brain Mapping: An Encyclopedic Reference* 79–90 (2015).
